# Regulators of *Trypanosoma brucei* Cell Cycle Progression and Differentiation Identified Using a Kinome-Wide RNAi Screen

**DOI:** 10.1371/journal.ppat.1003886

**Published:** 2014-01-16

**Authors:** Nathaniel G. Jones, Elizabeth B. Thomas, Elaine Brown, Nicholas J. Dickens, Tansy C. Hammarton, Jeremy C. Mottram

**Affiliations:** 1 Wellcome Trust Centre for Molecular Parasitology and Institute of Infection, Immunity and Inflammation, College of Medical, Veterinary and Life Sciences, University of Glasgow, Glasgow, United Kingdom; 2 Institute of Infection, Immunity and Inflammation, College of Medical, Veterinary and Life Sciences, University of Glasgow, Glasgow, United Kingdom; London School of Hygiene and Tropical Medicine, United Kingdom

## Abstract

The African trypanosome, *Trypanosoma brucei*, maintains an integral link between cell cycle regulation and differentiation during its intricate life cycle. Whilst extensive changes in phosphorylation have been documented between the mammalian bloodstream form and the insect procyclic form, relatively little is known about the parasite's protein kinases (PKs) involved in the control of cellular proliferation and differentiation. To address this, a *T. brucei* kinome-wide RNAi cell line library was generated, allowing independent inducible knockdown of each of the parasite's 190 predicted protein kinases. Screening of this library using a cell viability assay identified ≥42 PKs that are required for normal bloodstream form proliferation in culture. A secondary screen identified 24 PKs whose RNAi-mediated depletion resulted in a variety of cell cycle defects including in G1/S, kinetoplast replication/segregation, mitosis and cytokinesis, 15 of which are novel cell cycle regulators. A further screen identified for the first time two PKs, named repressor of differentiation kinase (RDK1 and RDK2), depletion of which promoted bloodstream to procyclic form differentiation. RDK1 is a membrane-associated STE11-like PK, whilst RDK2 is a NEK PK that is essential for parasite proliferation. RDK1 acts in conjunction with the PTP1/PIP39 phosphatase cascade to block uncontrolled bloodstream to procyclic form differentiation, whilst RDK2 is a PK whose depletion efficiently induces differentiation in the absence of known triggers. Thus, the RNAi kinome library provides a valuable asset for functional analysis of cell signalling pathways in African trypanosomes as well as drug target identification and validation.

## Author Summary

The African trypanosome, which is transmitted by the tsetse fly, causes the usually fatal disease Sleeping Sickness in humans and a wasting disease, called Nagana, in livestock in sub-Saharan Africa. There are no vaccines available against the diseases, and various problems are associated with current drug treatments (including toxicity to the patient and parasite drug resistance). Thus, it is important to identify essential parasite proteins that could be targeted by novel drugs. Protein kinases (PKs) are important cell signalling molecules, and are generally considered to have potential as drug targets. Here we report the construction of a library of trypanosome cell lines that allows us to specifically deplete each of the trypanosome's 190 PKs individually and analyse their function. Using this library, we show that ≥42 PKs are essential for proliferation of the mammalian-infective bloodstream form of the parasite (and thus have potential as drug targets), and demonstrate that 24 of these play important roles in coordinating cell division. We also shed light on how the parasite develops during its life cycle as it passes from the mammalian bloodstream form to the tsetse fly gut by identifying the first two PKs that regulate this life cycle developmental step.

## Introduction

The protozoan parasite, *Trypanosoma brucei*, is spread by the tsetse fly and causes both Human and Animal African Trypanosomasis in sub-Saharan Africa. It has a complex life cycle, whereby the parasite differentiates multiple times through a series of morphologically and biochemically distinct life cycle stages in each host. Some life cycle stages, such as the short stumpy bloodstream form (BSF) and the metacyclic form in the tsetse salivary glands are cell cycle arrested and pre-adapted for life in the new host, while others, such as the long slender BSF and the insect procyclic form (PCF) and epimastigote form proliferate. The signal transduction pathways regulating differentiation events are just starting to be unravelled. For example, differentiation of the long slender BSF to the short stumpy BSF occurs via a quorum-sensing pathway in response to an as yet unknown parasite-derived molecule termed Stumpy Induction Factor (SIF) [1]–[3]. A number of protein kinases (PKs), ZFK [4], MAPK5 [5] and TOR4 [6], have all been identified as negative regulators of this differentiation event, but the links between these kinases are not clear at present. Stumpy cells, which specifically express the citrate transporter PAD1 required to transduce a citrate and/or cis-aconitate signal [2], then differentiate to the PCF in the tsetse fly midgut, and a protein phosphatase cascade regulating stumpy to PCF differentiation has been described [7], [8], although the antagonising PKs are not yet known. A MAP Kinase Kinase, MKK1, has also recently been shown to regulate later differentiation steps in the tsetse fly, since *MKK1* null mutants are unable to establish salivary gland infections [9].

The cell division cycles of proliferative *T. brucei* stages are also complex, in part because of the need to faithfully replicate and segregate the parasite's several single copy organelles, and in part because there are key regulatory differences between *T. brucei* and mammalian cell cycles, as well as between different life cycle stages of the parasite. BSF *T. brucei* appear to have unusual mechanisms for regulating cell cycle checkpoints, as a block in mitosis inhibits cytokinesis but not successive rounds of nuclear DNA synthesis or kinetoplast replication and segregation [10]. A number of PKs have now been shown to regulate cell cycle progression in BSF *T. brucei*, for example, CRK1 and CRK3, which regulate G1/S and G2/M, respectively [11], AUK1 and TLK1, which are required for progression through mitosis [12]–[14], and PLK, PK50 and PK53 which regulate cytokinesis [15], [16], but many cell cycle/checkpoint regulators undoubtedly remain to be identified.

*T. brucei* has 158 eukaryotic PKs (ePKs), 12 predicted pseudokinases and 20 atypical PKs (aPKs), which phosphorylate proteins despite their lack of a conserved PK structure [17]–[19]. Although *T. brucei* does not possess any canonical receptor tyrosine kinase or tyrosine kinase-like kinase homologues, members of the other four major groups of ePKS, the AGC, CAMK, CMGC and STE kinases, are present, as are a number of ‘Other’ PKs. The CMGC, STE and Other/NEK groups of PKs are relatively expanded in *T. brucei* compared to humans. In the original analysis of the *T. brucei* kinome, additionally over 30 ‘Orphan’ PKs, which do not display sequence homology to any previously described PK families, were identified [17], although several of these have since been allocated to ePK groups [18], [19]. To date, 64 ePKs, 9 pseudokinases and 9 aPKs have been reported in at least one study to be essential for BSF proliferation, although some discrepancies do exist between different data sets, presumably reflecting the different methods employed to test essentiality. Some of these essential kinases have been identified via RNAi studies of individual genes [6], [11], [12], [12], [15,20], while others were identified via an RNAi screen of 30 PKs [21], an RNAi screen of CDXG kinases [22], a conditional null mutant screen of 20 targets [23] and a global RNAi target sequencing (RIT-seq) screen [24]. Here, we report the generation and screening of a global *T. brucei* kinome RNAi library of individual cell lines to identify essential PKs, cell cycle regulators and negative regulators of BSF to PCF differentiation.

## Results and Discussion

### Generation of the kinome RNAi library

In order to facilitate the high throughput generation of a library of individually targeted RNAi plasmids, the tetracycline-inducible, stem-loop RNAi vector, pRPa^ISL^ [25], was modified for recombineering. pRPa^ISL^ was selected because it integrates at a single, tagged locus resulting in high reproducibility between independent clones, and the modified vector, pGL2084 (Figure S1), allowed the generation of a stem-loop RNAi construct for a given gene in a single recombinase reaction. In total, RNAi constructs were created to target all 190 PK genes (Table S1). While the majority of RNAi constructs targeted a single PK, due to high levels of nucleotide sequence identity shared by some arrayed or paralogous PK genes, 6 plasmids each targeted two or more PKs. Each plasmid was linearised by digestion with *Asc*I and transfected individually into monomorphic *T. brucei* 2T1 BSF parasites [25]; at least two independent clones were recovered for further analysis.

### Screening the kinome RNAi library for protein kinases essential for proliferation *in vitro*

**Figure 1:**
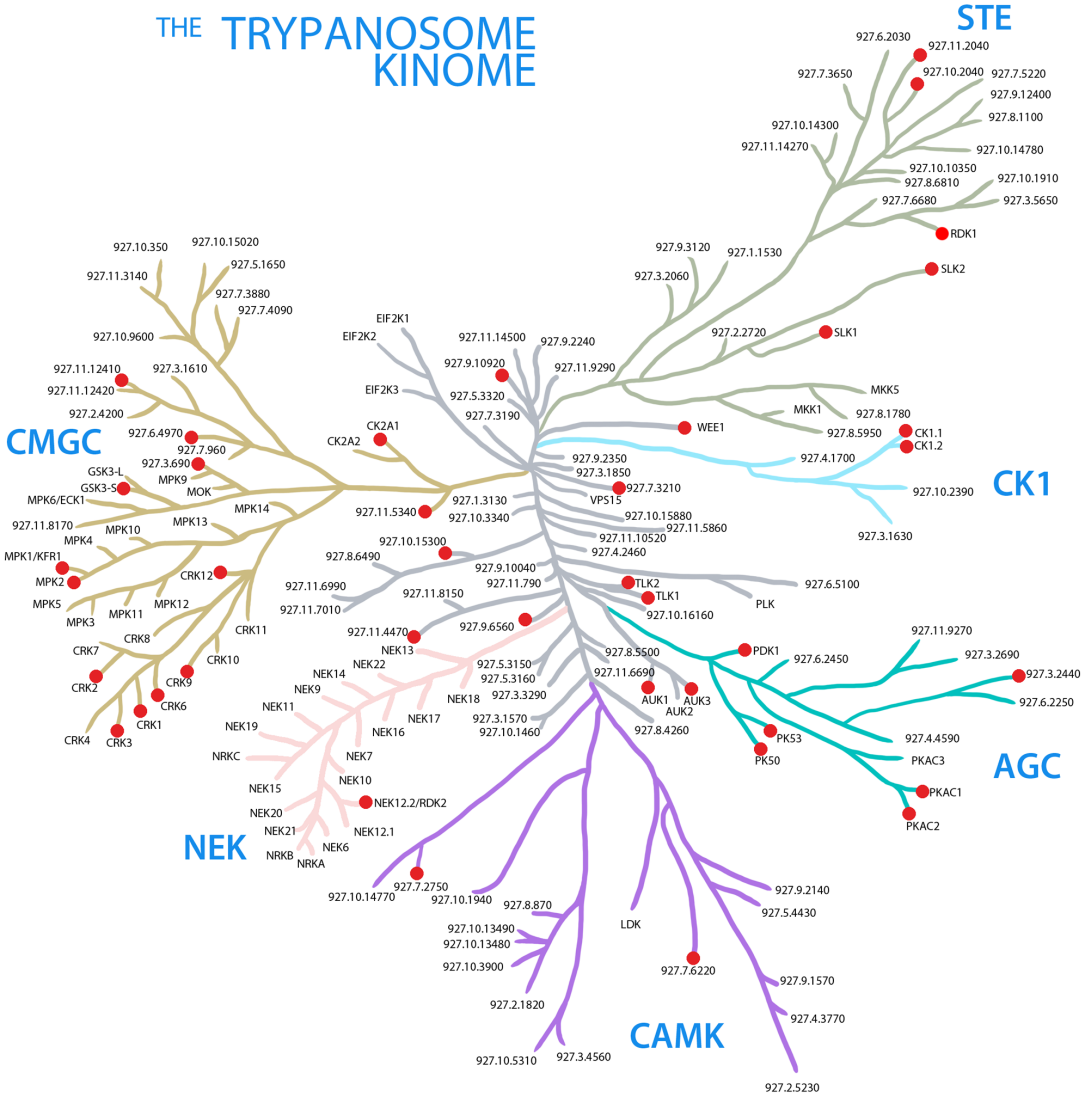
The trypanosome kinome. A stylised representation of the ePK complement in *T. brucei*. Protein kinases are grouped together into families defined by amino acid sequence similarities [51]. The node arrangements and lengths of the branches do not have any phylogenetic significance, and are for illustrative purposes only. Red nodes denote PKs, which, when depleted in this study, resulted in a loss of fitness phenotype.

**Figure 2:**
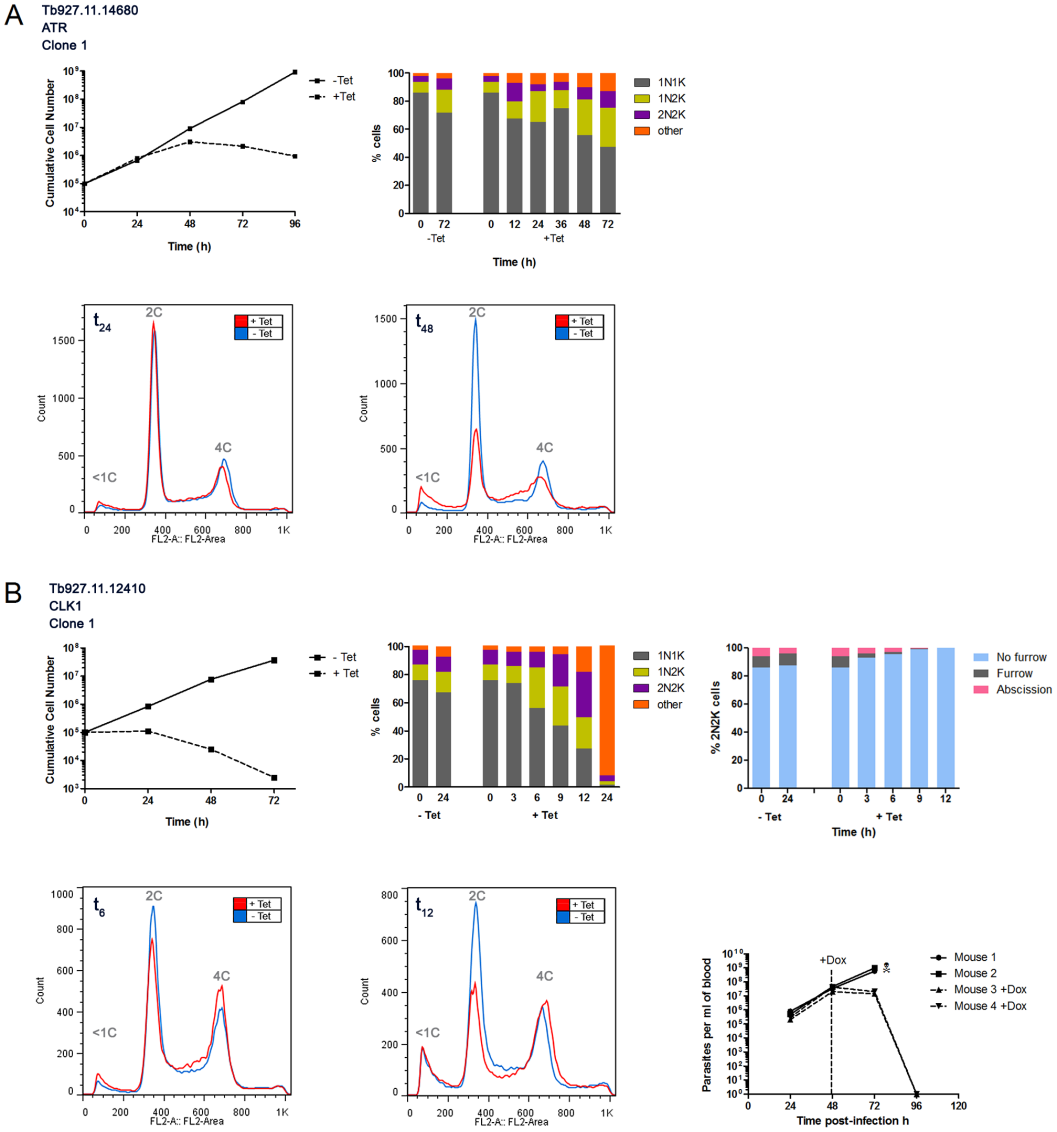
Protein kinases involved in the cell cycle. In vitro growth and cell cycle analysis for ATR (**A**) and CLK1 (**B**). **A**: Upper left: growth curves showing cumulative cell counts over time following tetracycline (Tet) induction (+) or not (−) of RNAi cell line in culture. Cell densities were maintained between 10^5^ and 10^6^ cells ml^−1^. Upper right: DAPI staining. Cells were stained with DAPI and the number of nuclei (N) and kinetoplasts (K) per cell was quantified (n>200) at the time-points indicated. Lower panel: flow cytometry profiles for 50,000 propidium iodide labelled cells at 24 hr or 48 hr post-induction. The DNA content of each peak is indicated. **B**: legend as for A, but including in upper right panel an analysis of the cytokinesis stage of 2N2K cells following tetracycline (Tet) induction (+) or not (−) of CLK1 RNAi cell line in culture. Lower left panels: flow cytometry profiles at 6 and 12 hours post-induction. Lower right: proliferation of CLK1 RNAi line in mice. 1×10^5^ trypanosomes were inoculated in 4 mice and RNAi was induced with doxycycline (Dox, as indicated) in 2 mice 48 hr later. Uninduced mice were culled as indicated (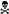) when their parasitaemias rose above 10^8^ cells ml^−1^.

To allow identification of essential kinases, a 72 hr cell viability assay using Alamar Blue was performed for two independent clonal cell lines for each gene. 48 cell lines (53 genes) showed loss of fitness (LOF) phenotypes (>10% reduction in Alamar Blue value in comparison with uninduced control values) after RNAi induction in at least one of the two biological replicates (Table S1). A secondary screen was performed on the LOF RNAi cell lines, examining their proliferation in culture over 96 hr following RNAi induction. At this stage, new cell lines were generated to knockdown CLK1 and CLK2, and NEK12.1 and NEK12.2 independently to facilitate subsequent characterisation of these kinases. In total, 42 cell lines (46 genes, including CLK1 and NEK12.2 as well as the STE11 kinase Tb927.11.14070 and TOR2, which had not exhibited a LOF phenotype by Alamar Blue assay when depleted (see below)) showed a growth defect (either slow growth (16 cell lines), growth arrest (15 cell lines) or cell death (11 cell lines)) under these conditions (Table 1, Figures 1, 2 and S2). Nine cell lines examined in the secondary screen did not display any significant growth defects upon RNAi induction (Figure S2A), including LDK1, depletion of which has previously been reported to result in only a minor growth defect in BSF *T. brucei* [26], and thus were not studied further here.

**Table 1:** Summary of the characteristics of RNAi cell lines displaying loss of fitness phenotypes as determined by Alamar Blue assay and growth curves.

Family	Name	GeneID	Growth defect	Cell cycle defect	Reference
AGC		Tb927.3.2440	Death	C	
AGC	PDK1	Tb927.9.4910	Slow	No	
AGC/NDR	PK50	Tb927.10.4940	Arrest	C	[16]
AGC/NDR	PK53	Tb927.7.5770	Slow	K/C	[16]
AGC/PKA*	PKAC1/PKAC2	Tb927.9.11100/Tb927.9.11030	Slow	C	[28]
CAMK		Tb927.7.6220	Arrest	C	
CAMK		Tb927.7.2750	Slow	No	
CK/CK1*	CK1.1/CK1.2	Tb927.5.790/Tb927.5.800	Death	K/C	[30]
CMGC		Tb927.11.5340	Slow	M	
CMGC/CDK	CRK1	Tb927.10.1070	Slow	G1/S	[11]
CMGC/CDK	CRK2	Tb927.7.7360	Slow	No	
CMGC/CDK	CRK3	Tb927.10.4990	Death	M	[11]
CMGC/CDK	CRK6	Tb927.11.1180	Slow	No	
CMGC/CDK	CRK9	Tb927.2.4510	Death	K	
CMGC/CDK	CRK12	Tb927.11.12310	Death	No	[23], [27]
CMGC/CLK	CLK1	Tb927.11.12410	Death	M/C	[22]
CMGC/GSK	GSK3-short	Tb927.10.13780	Arrest	M/C	[29]
CMGC/MAPK		Tb927.10.5140	Death	C	
CMGC/MAPK	KFR1	Tb927.10.7780	Slow	No	
CMGC/RCK		Tb927.3.690	Death	C	
CMGC/SRPK		Tb927.6.4970	Arrest	No	
Orphan	PK6	Tb927.9.10920	Arrest	No	
Other/AUR	AUK1	Tb927.11.8220	Death	M	[12], [13]
Other/AUR	AUK3	Tb927.9.1670	Slow	M/C	
Other/CAMKK		Tb927.10.15300	Slow	No	
Other/CK2	CK2A1	Tb927.9.14430	Arrest	K	
Other/NEK	NEK12.2/RDK2	Tb927.4.5310	Death	K/C	
Other/TLK*	TLK1/TLK2	Tb927.4.5180/Tb927.8.7220	Death	M/C	[14]
Other/ULK		Tb927.11.4470	Arrest	K/C	
Other/WEE	WEE1	Tb927.4.3420	Slow	No	
STE		Tb927.10.2040	Death	No	
STE/STE11		Tb927.11.2040	Arrest	No	
STE/STE11	RDK1	Tb927.11.14070	Slow	No	
STE/STE20	SLK1	Tb927.8.5730	Arrest	No	
STE/STE20	SLK2	Tb927.9.12880	Arrest	No	
Pseudo Other/NAK		Tb927.9.6560	Arrest	No	
Pseudo Orphan		Tb927.7.3210	Slow	U	
aPK/PIKK	ATR	Tb927.11.14680	Arrest	M	
aPK/PIKK	TOR1	Tb927.10.8420	Slow	U	[20]
aPK/PIKK	TOR2	Tb927.4.420	Death	No	[20]
aPK/PIKK	TOR4	Tb927.1.1930	Slow	G1/S	[6]
aPK/RIO	RIO1	Tb927.3.5400	Arrest	No	
aPK/RIO	RIO2	Tb927.6.2840	Arrest	No	

C, cytokinesis; M, mitosis; K, kinetoplast duplication/segregation; U, unclassified. Asterisks indicate RNAi cell lines targeting ≥1 PK.

Amongst the 45 genes whose depletion results in a LOF phenotype were members of all the PK families represented in *T. brucei* (see Table 1 for gene IDs). Fifteen of these PKs had been studied previously and reported either to be essential for BSF *T. brucei* proliferation *in vitro*, or to be required for optimal growth. These were the AGC family kinases, PK50 and PK53 [16], the CMGC family cdc2-related kinases, CRK1, CRK3 and CRK12 [11], [27], the cdc2-like kinase CLK1 [22], the PKA catalytic subunits, PKAC1 and PKAC2 [28], GSK3-short [29], the Aurora kinase, AUK1 [12], [13], the tousled-like kinase, TLK1 [14], CK1.2 [30] and the TOR kinases, TOR1, TOR2 and TOR4 [6], [20].

Twenty other PKs reported in a previous global RITseq RNAi screen [24] to be essential for BSF growth were also detected here (Table S1). These include CRK9, MPK2, and CK2A1. In addition, depletion of TbAUK3, in contrast to an earlier study [12], or down-regulation of the tousled-like kinase, TLK2, generated substantial growth defects. A ULK homologue (Tb927.11.8150), a potential regulator of autophagy [31], was also observed to result in a moderate growth defect upon RNAi ablation in this study. Other RITseq ‘hits’ confirmed by this study include the AGC kinase, Tb927.3.2440, the CMGC/RCK kinase, Tb927.3.690, the CAMKs Tb927.7.2750 and Tb927.8.870, the CAMKK, Tb927.10.15300, the orphan kinase, PK6, NEK12.2 and two members of the pseudokinase family (Tb927.9.6560 and Tb927.7.3210).

Fourteen PKs were shown here to be important for the proliferation of BSF *T. brucei* in culture for the first time. These include the AGC kinase Tb927.9.4910, which contains a PK domain most similar to PDK1, a master AGC kinase in other eukaryotes [32] the CAMK Tb927.7.6220, a number of CMGC kinases: CRK2, CRK6, the MAPK, KFR1, and a serine-arginine-rich protein kinase (Tb927.6.4970), as well as an undefined CMGC PK (Tb927.11.5340) and WEE1. Several STE family members, which are potential upstream activators of the MAPKs, were also newly identified as playing important or essential roles in cell growth, including two STE11-type PKs (Tb927.11.2040 and Tb927.8.1100), the STE20 family MAPKKK, SLK1, and an unclassified STE kinase, Tb927.10.2040. Three atypical protein kinases, ATR and RIO1, were also shown for the first time to be important for cell growth.

Overall, the loss of fitness phenotypes detected in our kinome-wide RNAi screen has an excellent correlation with previous studies knocking down individual genes (Tables 1 and S1). Additionally, two protein kinases shown previously to be non-essential for growth by gene knockout, ZFK [4] and MKK1 [33], did not show a loss of fitness phenotype in our screen. However, one notable discrepancy with the published literature was PLK, which has been shown to be essential in BSF [15], but gave no RNAi phenotype in this study. qPCR analysis, however, revealed that in the two cell lines used in this study, no reduction in *PLK* mRNA was detected after RNAi induction, explaining the lack of the expected phenotype (Figure S3). To monitor mRNA knockdown more widely, qPCR was performed for a number of target genes, encompassing cell lines with both loss of fitness phenotype and no phenotype upon RNAi induction. Transcripts were substantially reduced (typically by 40–60%) for 11 of the 12 other genes studied by qPCR (Figure S3). For *MKK5*, qPCR indicated there to be inefficient knockdown of mRNA. While MKK5 is known to be redundant in BSF parasites [33], this does raise the possibility that other false negatives arising from inefficient mRNA knockdown may be present within the screen dataset.

Correlation with the genome wide RITseq screen of Alsford [24] was also good, with 70% of the individually targeted genes giving the same 3 day growth phenotype. A severe growth phenotype (defect apparent at 3 days post-RNAi induction) was detected for 20 PKs by Alsford, but not in this study (Table S1). In contrast, this study identified 25 PKs whose depletion resulted in a growth defect that were not identified by Alsford (Table S1). The variance between the two studies is likely to have arisen through the use of different strains of *T. brucei*, different RNAi constructs and different methods for assessing cell growth, highlighting the importance of using complementary approaches in such studies.

### Screening protein kinases important for BSF proliferation for cell cycle defects

The 42 RNAi cell lines displaying a growth defect in culture were analysed using DAPI staining to identify PKs with a potential role in cell cycle control (see Tables 1, S2, Figures 2, S2 B–G). The nucleus and kinetoplast undergo discrete replication cycles in *T. brucei*, with the kinetoplast commencing replication slightly ahead of the nucleus. A cell in G1 phase has 1 nucleus and 1 kinetoplast (1N1K cell), while a cell in G2/M phase will have 1 nucleus and 2 kinetoplasts (1N2K) and a cell that has undergone mitosis but has not yet undergone cytokinesis will have 2 nuclei and 2 kinetoplasts (2N2K). Any other NK configuration is abnormal. Growth curves were used to determine the optimal time points for performing DAPI staining (before, during and after the appearance of the growth defect) for each cell line and flow cytometry was used to confirm the DAPI cell cycle analysis for specific cell lines (Figures 2 and S2H). Eighteen cell lines, including CRK2, CRK6 and CRK12 [11], [27], did not exhibit any obvious cell cycle defects, or only displayed cell cycle changes after the emergence of the growth defect, suggesting that these kinases are unlikely to play a direct or specific role in cell cycle regulation (Tables 1, S2, Figure S2 B,D,F).

Twenty-four cell lines displayed cell cycle defects following RNAi induction (Tables 1, S2, Figures 2, S2 C, E, G). The phenotypes obtained with six of these cell lines were consistent with published literature: PK50 [16], PKAC1/PKAC2 [28], CRK1 and CRK3 [11], AUK1 [12], [13] and TOR4 [6], while two, including TOR1 whose depletion has previously been reported to arrest cells in G1 [20], showed phenotypes that could not be classified due to the lack of a pattern in aberrant cell types. It is possible that TOR1 was knocked down less efficiently in our cell line, since we only observed slow growth rather than growth arrest following RNAi induction. Similarly, with PK53, a different phenotype was observed than reported previously [16]; induced RNAi cells displayed slower growth rather than cell death, and rather than depletion of PK53 blocking cells during cytokinesis furrowing, increased numbers of 2N1K cells were observed, suggesting that PK53 depletion affected kinetoplast and/or basal body replication and/or segregation. Such a discrepancy might be explained by differing levels of PK53 knockdown in the two studies. PKs targeted by the remaining 15 cell lines were identified as novel cell cycle regulators (Tables 1, S2, Figures 2, S2). Several of these kinases appear to play roles in kinetoplast division. Depletion of CRK9 or CK2A1 resulted in a reduction in 1N2K and 2N2K cells, accompanied by an increase in abnormal cell types, of which the most abundant were 2N1K cells (13% and 19% total cells for CRK9 and CK2A1 RNAi, respectively, at 48 hours post-induction), suggesting defects in kinetoplast replication or segregation. Previously, CRK9 was reported to affect mitosis and cytokinesis in PCF *T. brucei*, but no phenotype was detected upon depletion of CRK9 in the BSF [34].

Depletion of CK1.1/CK1.2 resulted in dramatic increases in 2N2K and abnormal cells from 20 hours post-induction. The most abundant abnormal cells were 2N1K cells, which comprised 16% of total cells at 24 hours post-induction. The remainder of the abnormal cells had a variety of N/K configurations (including >2N>2K cells), but no zoids were detected. Taken together, these data suggest down-regulation of CK1.2 (CK1.1 is redundant in BSF *T. brucei*) may affect kinetoplast division and cytokinesis, adding to existing data showing that this kinase is essential [30]. Similarly, depletion of NEK12.2 resulted in increased 2N2K and abnormal cells (of which the majority were 2N1K cells with no zoids detected) from 35 hours post-induction, suggesting that this kinase also (in addition to a role in BSF to PCF differentiation described below) regulates kinetoplast division and cytokinesis. Down-regulation of Tb927.11.4470 led to increased 2N2K and abnormal cell types (2N1K as well as cells with multiple nuclei and/or kinetoplasts (>2N>2K cells)) at 48 hrs post-induction, potentially indicating a role for this kinase in kinetoplast replication/segregation and/or cytokinesis. Two kinases, the CMGC kinase, Tb927.11.5340, and ATR (Figure 2A) appear to be mitotic regulators, since their depletion resulted in increased 1N2K and 1N>2K cells (more dramatic upon ATR RNAi where these cell types comprised 33% and 15% of the total cells, respectively, at 48 hours post-induction). Flow cytometry indicated that DNA replication had occurred in these cells (Figure S2H), suggesting that progression through mitosis was inhibited, invoking the mitosis to cytokinesis checkpoint [35] allowing kinetoplast re-replication in the absence of cell division. Four kinases, the AGC kinase Tb927.3.2440, the CAMK, Tb927.7.6220, MPK2 and the CMGC/RCK kinase Tb927.3.690 were identified as being essential for cytokinesis, since their depletion resulted in the accumulation of 2N2K and >2N>2K aberrant cell types. Depletion of Tb927.3.2440 and Tb927.3.690 resulted in the most dramatic phenotypes, with almost all cells being abnormal by 48 hours post-induction.

Additionally, four PKs (GSK3-short, AUK3, TLK2 and CLK1) may play roles in either or both mitosis and cytokinesis. GSK3-short RNAi resulted in slight increases in 1N2K and 2N2K cells and the appearance of small numbers of ‘other’ abnormal cell types (including 2N1K and 0N1K cells), and only minor changes to the flow cytometry profile (Figure S2H), suggesting DNA replication proceeded normally followed by a possible delay in mitosis and/or defects in cytokinesis. Previous studies of GSK3-short have shown it to be essential for viability in BSF *T. brucei* and validated it as a potential drug target, but have not examined its cell cycle phenotype [29]. RNAi of AUK3 resulted in large numbers of ‘other’ cell types appearing from 18 hours post-induction. The most common types of abnormal cells were 2N1K and 0N1K cells, together comprising ∼70% and ∼60% of the abnormal cell population at 18 and 24 hours, respectively. Also prevalent were cells with multiple nuclei and/or kinetoplasts (>2N>2K; 17% and 31% at 18 and 24 hours, respectively). Thus, it is possible that AUK3 is involved in mitosis and/or cytokinesis since a delay in mitosis or defects in furrow ingression during cytokinesis could result in the generation of these cell types [35].

Depletion of TLK1/TLK2 resulted in a large increase in 1N2K and 0N1K cells, reaching 43% and 13%, respectively, at 36 hours post-induction, concomitant with a reduction in 2N2K cells. Flow cytometry revealed an increase in the <1C peak and a decrease in the 2C peak, consistent with the increase in 0N1K cells and decrease in proportion of 1N1K cells, respectively, and a shift of the 4C DNA content peak to the right, indicating that many cells (presumably 1N2K and ‘others’) were aneuploid and had over-replicated their DNA (Figure S2H). Previously, TLK1 depletion in BSF was shown to cause defective spindle formation and chromosome segregation, resulting in an increase in 1N2K and abnormal cells (but not zoids), but did not affect 2N2K cell numbers [36]. While TLK2 depletion did not result in any observable phenotype in PCF *T. brucei* [14], it is possible that it does play a role in BSF, accounting for the differences in phenotype between the TLK1/TLK2 double knockdown and TLK1 single knockdown cell lines. Thus, in BSF *T. brucei*, it is possible that knockdown of TLK1 and TLK2 inhibits mitosis but does not trigger the mitosis to cytokinesis checkpoint, resulting in inappropriate cytokinesis in 1N2K cells leading to the formation of zoids (0N1K cells). This would imply that TLK2 is involved in this checkpoint. Given that many cells were aneuploid, it may be that cells reinitiated DNA synthesis (but did not complete it) prior to cytokinesis occurring in 1N2K cells.

Depletion of CLK1 resulted in a dramatic increase in 1N2K, 2N2K and abnormal cell types from 6–9 hrs post-induction (Figure 2B). At 9 hours post-induction, the majority of the abnormal cell types had configurations of 2N3K or 2N4K, but at later timepoints, the N/K configurations of many cells became difficult to accurately determine since discrete nuclei and kinetoplasts could no longer be seen. Flow cytometry revealed a decrease in the 2C peak and an increase in the 4C peak (Figures 2 and S2), consistent with an accumulation of cells that had replicated their DNA. These data suggest mitosis and cytokinesis, but not DNA replication, were deregulated in these cells. To investigate the possible cytokinesis defect further, the cytokinesis stage of >200 2N2K cells was determined up to 12 hours post-induction, showing that the majority of the accumulated 2N2K cells had no furrow and were therefore yet to commence cytokinesis. Thus, CLK1 appears to be required for mitosis and entry into cytokinesis and is also essential for survival of trypanosomes in a mouse model, thereby validating CLK1 as a potential drug target [22].

In summary, cell cycle screening of our kinome RNAi library has allowed putative cell cycle functions to be assigned to 15 PKs for the first time, although further, more detailed analyses will be required to confirm these functions. However, the fact that we were also able to reproduce a number of RNAi phenotypes obtained for PKs studied previously provides a level of confidence in the screen findings. The simple cell cycle screen applied here was also sensitive enough to be able to pick up defects across the whole cell cycle from G1/S to kinetoplast division to mitosis and cytokinesis. Since additional simple screens e.g. using immunofluorescence to investigate replication of organelles and/or structural components of the trypanosome cell or flow cytometry to investigate DNA replication could be applied to identify the PKs responsible, this library adds to the tool-kit for the study of the *T. brucei* cell cycle.

### Identification of a STE11-like ePK that represses bloodstream to procyclic form differentiation

**Figure 3:**
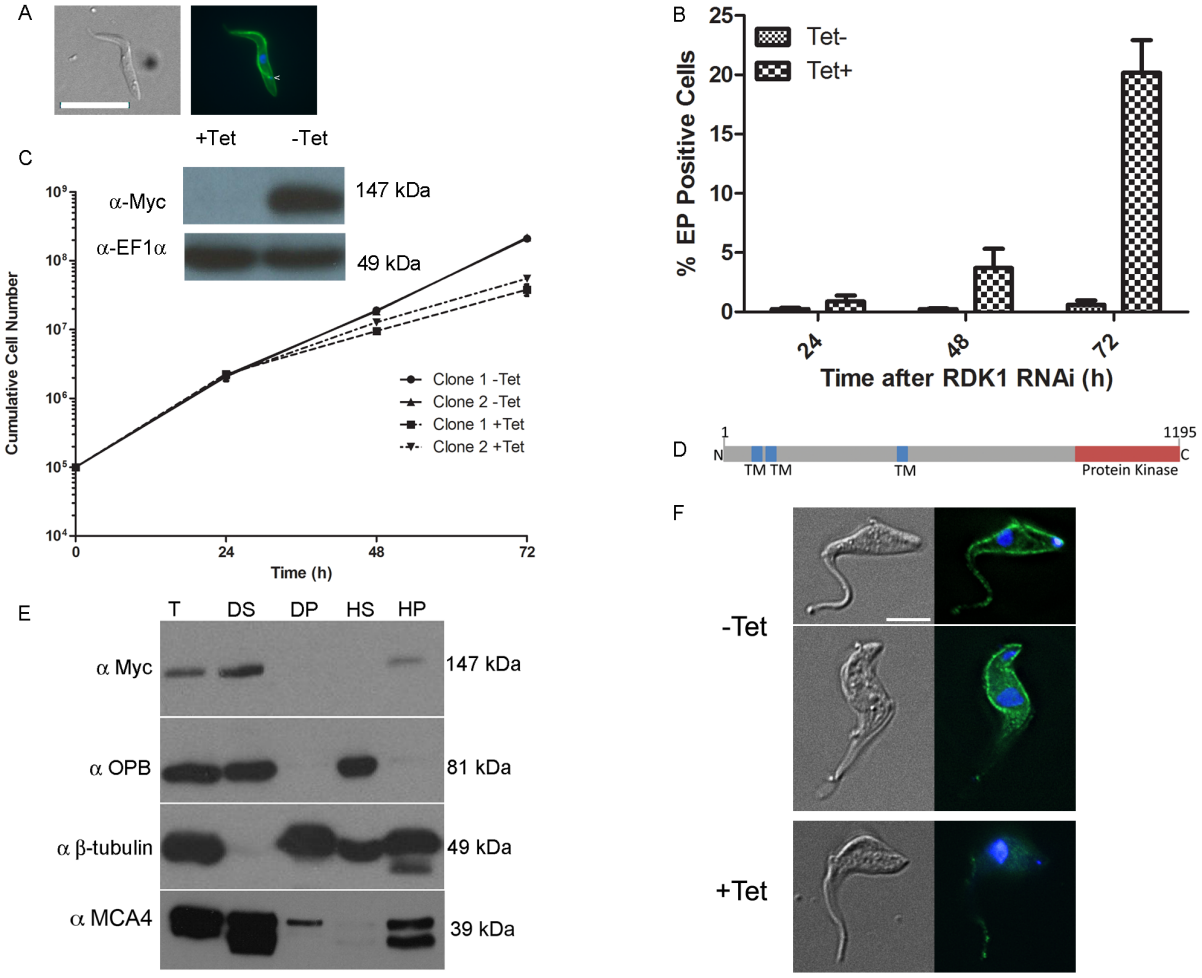
RDK1 is a repressor of BSF to PCF differentiation. **A**: *RDK1* RNAi was induced for 72 hours before cells were analysed by (immuno)fluorescence microscopy with DAPI (to stain nuclei and kinetoplasts) and FITC-conjugated anti-EP procyclin. Left panel: DIC images; right panel: merged fluorescence channels - DAPI (blue), EP-procyclin (green). Scale bar: 10 µm. **B**: quantification of EP procyclin-expressing cells by flow cytometry over time following induction of RDK1 RNAi. **C**: Growth curves showing cumulative cell counts over time following tetracycline (Tet) induction (+) or not (−) of *RDK1* RNAi in culture. Cell densities were maintained between 10^5^ and 10^6^ cells ml^−1^. Error bars indicate the standard deviations around the means of three technical replicates. Inset: Analysis of RDK1 protein knockdown following RNAi induction. *RDK1* RNAi cell line clone 1 expressing *RDK1::12Myc* from the endogenous locus was analysed by Western blotting with an anti-Myc antibody 24 hr after RNAi induction. Anti-EF1α antibody was used as a loading control. **D**: predicted domain structure of RDK1. TM: transmembrane domain. **E**: cell fractionation. Cells expressing RDK1::12myc were fractionated and analysed by Western blotting with anti-Myc antibody to detect RDK1::12myc and anti-OPB, anti-β-tubulin and anti-MCA4 antibodies as cytoplasmic, cytoskeletal and membrane protein controls, respectively. T: total cell lysate; DS: detergent-soluble; DP: detergent-pellet; HS: hypotonic soluble; HP: hypotonic pellet. F: immunofluorescence of RDK1::12Myc expressed in an RDK1 RNAi background. Cells were induced (+) or not (−) with tetracycline (Tet) for 22 hr and fixed, permeabilised and labelled with DAPI (blue) and an anti-Myc antibody (green). Left: DIC image; right: DAPI/Myc merge. Scale bar: 5 µm.

Differentiation of *T. brucei* BSF parasites to PCF parasites involves major changes in gene and protein expression, metabolism and morphology (reviewed in [3]). A protein phosphatase cascade has been shown to regulate the differentiation of stumpy BSF parasites to PCF parasites [7], [8], but to date, no protein kinases regulating this process have been identified. Here, PKs involved in the negative regulation of differentiation were sought by microscopic evaluation of cells 72 hr after tetracycline induction, looking for morphological changes consistent with differentiation from the BSF to the PCF [37]. Following induction, the presence of cells with PCF-like morphology was detected for the STE11-like PK, Tb927.11.14070, RNAi cell line which had not shown a noticeable loss of fitness in the Alamar Blue screen. Immunofluorescence analysis showed that RNAi of Tb927.11.14070led to expression of the PCF–specific surface protein, EP procyclin [37], and that the kinetoplast repositioned to midway between the posterior of the cell and the nucleus, in a manner characteristic of PCF cells (Figure 3A). Following RNAi induction and growth at 37°C, the proportion of EP procyclin positive cells reached 20% by 72 hr, as assessed by flow cytometry (Figure 3B), which is consistent with the percentage of differentiation competent stumpy-like parasites (stumpy*) in the monomorphic 2T1 cell line [25]. These data indicate that this PK, named Repressor of Differentiation Kinase 1 (RDK1), is a repressor of BSF to PCF differentiation; depletion of RDK1 allows differentiation-competent cells to proceed to the procyclic form even at 37°C, the temperature of the mammalian bloodstream which is higher than that found during natural transmission in the tsetse (nominally 27°C). To investigate further the effect of RDK1 depletion, growth curves were performed for RDK1 RNAi lines cultured *in vitro* at 37°C. Slow growth was detected from 48 hr post-induction for RDK1 RNAi lines (Figure 3C). Knockdown of RDK1 was confirmed at the mRNA level by qPCR (Figure S3) and at the protein level by expressing a C-terminal 12-Myc tagged version of RDK1 (RDK1::12myc) from the endogenous locus and Western blotting (Figure 3C). RDK1 is predicted (using the TMHMM algorithm) to contain three transmembrane domains in the N-terminal domain, with the PK domain at the C-terminus (Figure 3D) suggesting that RDK1 is a membrane protein. The third predicted transmembrane domain corresponded to a region predicted to be disordered and may be a false positive; therefore, the number and topology of the TM domains remains unclear. Cell fractionation analysis showed that RDK1::12myc was associated with the membrane fraction (Figure 3E) as RDK1::12Myc was found in the supernatant fraction of a detergent based lysis, which contains membrane and cytoplasmic components, and in the pellet fraction of a hypotonic lysis which contains membrane and cytoskeletal proteins. This fractionation pattern was the same as that observed for MCA4, a protein previously identified as cell membrane associated [38]. Immunofluorescence microscopy was performed with an anti-Myc antibody to detect RDK1::12Myc in an RDK1 RNAi background (see above). In uninduced cells, RDK1::12Myc staining outlined the cell body and was also present on the flagellum, although often the flagellum staining was much weaker than the cell body staining (Figure 3F). Upon induction of RDK1 RNAi, the RDK1::12Myc signal outlining the cell body disappeared by 22 hours post-induction, and the flagellum staining was much reduced. This might suggest that RDK1 is turned over more slowly in the flagellum than in the rest of the cell. These data support a cell membrane location for RDK1, which correlates with the identification of RDK1 in the flagellum surface proteome and the annotation of RDK1 as a cell surface receptor kinase [39].

In order to provide further evidence that the cells were differentiating to true PCF parasites, rather than simply remodelling their morphology and cell surface, the transcriptome of the RDK1 RNAi induced cells was examined by RNA-Seq analysis. At 48 hr post-induction, mRNA was extracted from induced and uninduced RDK1 RNAi cell lines and sequenced. Analysis of differentially regulated transcripts identified a total of 479 genes whose expression was significantly different in the induced and uninduced samples, of which 275 were identified in both biological replicates. 145 of these were down-regulated (Table S3). These were compared with genes previously identified as being differentially expressed in BSF or PCF trypanosomes by microarray or RNA-Seq [40], [41]; 236 of the 275 differentially expressed genes detected in RDK1 RNAi induced cells were also identified as differentially expressed genes in these studies, including procyclic form-specific proteins, EP- and GPEET- procyclin, and trans-sialidase and BSF specific proteins ESAG and ISG64. Hence, despite only 5% of cells having morphological and cell surface signatures of procyclic form parasites at 48 hours post-induction, the transcriptome data support a BSF to PCF differentiation process occurring after ablation of RDK1.

**Figure 4:**
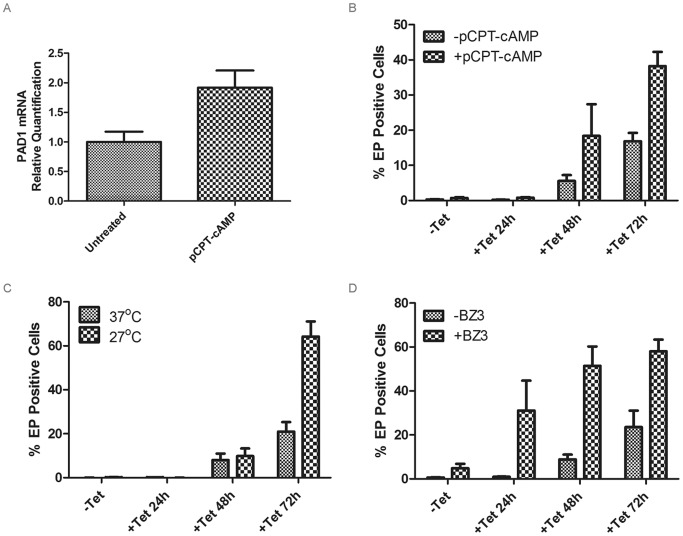
Analysis of differentiation during RDK1 RNAi. **A**: qRT-PCR analysis of PAD1 mRNA expression after 24 hr treatment of uninduced RDK1 RNAi cells with 250 µM 8-pCPT-cAMP. **B–D**: quantification of EP procyclin-expressing cells by flow cytometry following induction of RDK1 RNAi. RNAi of RDK1 was induced for 24, 48 and 72 hr by addition of tetracycline (Tet) and the percentage of EP-procyclin positive cells detected by flow cytometry after treatment with 250 µM 8-pCPT-cAMP (**B**), cold shock treatment of cells at 27°C (**C**) or incubation with 150 µM BZ3 (**D**) for the final 24 hr of each RNAi induction.

The *T. brucei* 2T1 monomorphic cell line becomes more responsive to signals of differentiation when treated with cell permeable, hydrolysable cAMP analogues [42], as the parasites undergo cell cycle arrest and express stumpy-specific genes such as PAD1 [1], [3], [42]. To test if RDK1 acts in a signalling pathway that is parallel to, or downstream of, SIF, the RDK1 RNAi line was treated with 250 µM 8-(4-chlorophenylthio)-cAMP (8-pCPT-cAMP). Incubation with 8-pCPT-cAMP led to a 2-fold increase in expression of the stumpy gene *PAD1*, which encodes a carboxylate surface transporter (Figure 4A), suggesting an increase in differentiation-receptive stumpy* parasites. When 8-pCPT-cAMP treatment was applied to cells depleted of RDK1, the percentage of EP procyclin positive cells increased ∼2-fold from 20% to 38% after 72 hr (Figure 4B), indicating an additive response to the depletion of RDK1 and the induction of stumpy* formation. The effect was even more pronounced following 24 hr cold shock at 27°C, when 60% of cells were EP procyclin positive in the induced cell line (Figure 4C). Similarly, when the PTP1 phosphatase inhibitor BZ3 [7] was incubated with the RDK1 RNAi induced cell line, the proportion of EP procyclin positive cells increased from 20% to 60% after 72 hr, indicating an additive response to the depletion of RDK1 and the inhibition of PTP1 (Figure 4D). These data suggest that the protein kinase, RDK1, and the tyrosine phosphatase, PTP1, are working together as repressors of differentiation in discrete signalling pathways that respond to the SIF differentiation signal. RDK1 has some sequence identity with STE11-like MAP kinase kinase kinases, which operate with STE5 scaffold proteins to phosphorylate STE7 PKs within a MAP kinase signalling cascade. No STE5 proteins have been identified in trypanosomes, raising the possibility that the 2 or 3 trans-membrane domains of RDK1 act to anchor the protein in the membrane and initiate a MAP kinase signalling cascade that represses differentiation of BSF to PCF; such a pathway would be likely to operate in association with the PTP/PIP39 phosphatase cascade [3], [7], [8].

### Identification of a NEK family ePK that represses bloodstream to procyclic form differentiation

**Figure 5:**
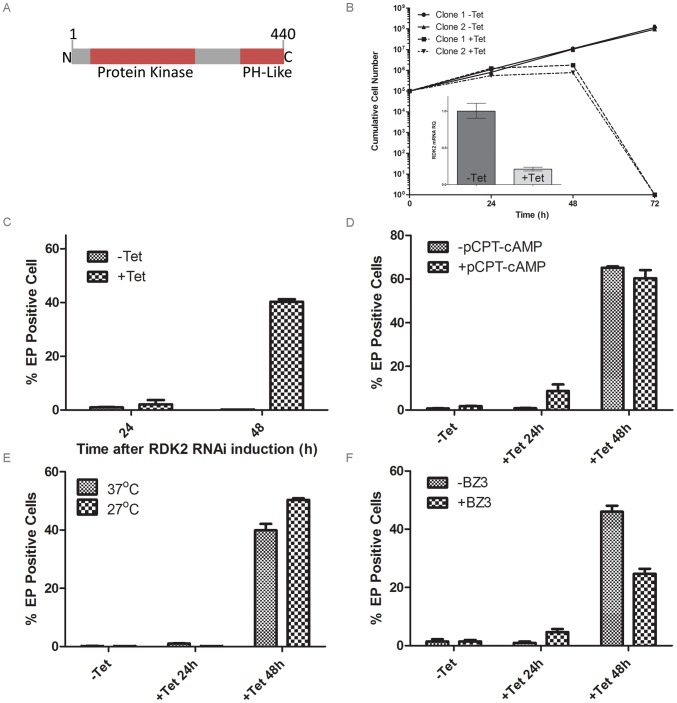
RDK2 is a repressor of BSF to PCF differentiation. **A**: Predicted domain structure of RDK2. PH: pleckstrin homology domain. **B**: Growth curves showing cumulative cell counts over time following tetracycline (Tet) induction (+) or not (−) of *RDK2* RNAi in culture. Cell densities were maintained between 10^5^ and 10^6^ cells ml^−1^. Error bars indicate the standard deviations around the means of three technical replicates. Inset: qRT-PCR analysis of RDK1 mRNA knockdown following RNAi induction. **C**: quantification of EP procyclin-expressing cells by flow cytometry following induction of RDK2 RNAi. RNAi of RDK2 was induced for 24 and 48 hr by addition of tet and the percentage of EP-procyclin positive cells detected by flow cytometry after treatment with 250 µM 8-pCPT-cAMP (**D**), cold shock treatment of cells at 27°C (**E**) or incubation with 150 mM BZ3 (**F**) for the final 24 hr of each RNAi induction.

The NEK family of PKs is highly expanded in number in *T. brucei* in comparison with humans, and the family was predicted to contain protein kinases with parasite-specific functions. Simultaneous depletion of two closely related NEK kinases, NEK12.1 and NEK12.2 (Tb927.8.7110 and Tb924.4.5310), resulted in a severe growth phenotype both in culture and in a mouse model (Figure S4), making one or other or both of these NEK kinases potential drug targets. NEK12.1 is unusual as it is one of only two *T. brucei* ePKs to possess a small gatekeeper residue (A117) in its ATP binding pocket; NEK12.2 possesses a bulkier methionine gatekeeper residue (M117). This suggests that the active site pocket of NEK12.1 is larger than that of NEK12.2 and other active PKs in *T. brucei*, and is likely to be able to accommodate bulky ATP inhibitors that do not fit within the ATP binding pocket of PKs with bulkier gatekeeper residues [43]. NEK12.2 has an N-terminal protein kinase domain and a pleckstrin homology (PH)-like domain at the C-terminus (Figure 5A). The *NEK12.1* and *NEK12.2* paralogues share 87% DNA sequence identity, and their high sequence similarity and location on chromosomes 8 and 4, respectively, mean that they may have resulted from a chromosome translocation [44].

To investigate the role of each kinase, individual NEK12.1 and NEK12.2 RNAi cell lines were generated by creating RNAi target sequences through the joining of short DNA sequences (≥20 nt) taken from small, divergent regions spread across each gene. Induction of NEK12.2 RNAi resulted in cell death after 72 hr (Figure 5B), whilst NEK12.1 grew at the same rate as the uninduced cell line (not shown). qPCR confirmed mRNA knockdown for NEK12.1 and NEK12.2 in their respective RNAi lines (Figure 5B, Figure S3, Figure S4). NEK12.1 mRNA was also observed to decrease under NEK12.2 RNAi induction; however, as the RNAi sequences targeted equivalent regions of each gene and NEK12.2 mRNA was not observed to decrease after NEK12.1 RNAi induction, cross targeting was not thought to be occurring. Instead, it was suspected (see below) that NEK12.2 RNAi triggers a spontaneous PCF differentiation phenotype similar to the one observed in RDK1 RNAi cell lines, and NEK12.1 mRNA has been shown previously to be down-regulated in differentiating parasites and PCF parasites compared to BSF [41], [45]. These data suggest that NEK12.2 is an essential PK and may play a role in parasite differentiation, which was investigated further.

Following NEK12.2 depletion, the morphology of the cells appeared PCF-like, so immunostaining for EP procyclin was conducted. When analysed by flow cytometry, 40–60% of cells were EP procyclin positive after 48 hr of NEK12.2 RNAi induction (Figure 5C–F), demonstrating a more rapid and more severe phenotype than observed in induced RDK1 RNAi cells. Immunofluorescence microscopy showed that the EP procyclin positive cells had repositioned their kinetoplast to midway between the posterior of the cell and the nucleus (not shown). These data indicate that NEK12.2 (renamed RDK2) is also a repressor of BSF to PCF differentiation. In contrast to RDK1 RNAi, incubation of the induced RDK2 RNAi line with 8-pCPT-cAMP or subjecting it to cold shock (27°C) did not potentiate the differentiation phenotype (proportion of EP procyclin positive cells) (Figure 5D, 5E), indicating that RDK2 depletion is sufficient to induce differentiation of the entire sub-population of bloodstream form cells that is sensitive to these differentiation triggers. Additionally, treatment of induced RDK2 RNAi cells with BZ3 did not increase the proportion of EP procyclin positive cells (Figure 5F); rather, BZ3 treatment actually resulted in a decrease in such cells, hinting at a possible negative feedback mechanism. While the cell death phenotype associated with RDK2 RNAi (most likely due to the observed cell cycle defects in kinetoplast division and cytokinesis, see above) complicates the interpretation of these data and prevented analysis of the differentiation phenotype over a longer time period, the efficiency with which RDK2 depletion induces differentiation in the absence of other triggers suggests RDK2 plays a vitally important role in controlling BSF to PCF differentiation.

### Concluding remarks

This study demonstrates that it is feasible to perform large scale functional analysis of gene families, since the recombineering-based system used to generate the library of RNAi plasmids has proven to be efficient, robust and rapid. This should facilitate RNAi studies on other large gene families, or even the complete genome. Additionally, the kinome library of RNAi cell lines itself is an important resource, since it permits systematic and global analysis of PK signalling in *T. brucei*. The ability to use the 2T1 derived cell lines with endogenous tagging constructs or other expression cassettes further allows the generation of reporter cell lines to interrogate the role of each PK in controlling any cellular process of interest.

This study confirms the importance of protein phosphorylation to the parasite [46]; while the absence of a proliferation phenotype upon RNAi induction does not provide sufficient evidence that a gene is non-essential, this study has identified at least 42 (considering the double knockdown cell lines) out of 190 PKs (22.1%), to be required for normal BSF proliferation. 13 of these PKs are worthy of prioritisation as drug targets, as RNAi induced cell lines die very rapidly upon RNAi induction. These include the CMGC PKs CRK3, CRK9, CRK12, CLK1, Tb927.10.5140 and Tb927.3.690, the AGC PK Tb927.3.2440, CK1.1, AUK1, RDK2, TLK, TOR2 and the STE PK Tb927.10.2040. Chemical proteomic profiling has shown that *T. brucei* PKs are sensitive to inhibitors with nM potency [18] and hypothemycin, a fungal natural product with anti-trypanosomal activity, inhibits both GSK3short and CLK1 [22], providing support for the concept that trypanosome-specific inhibitors can be developed.

**Figure 6:**
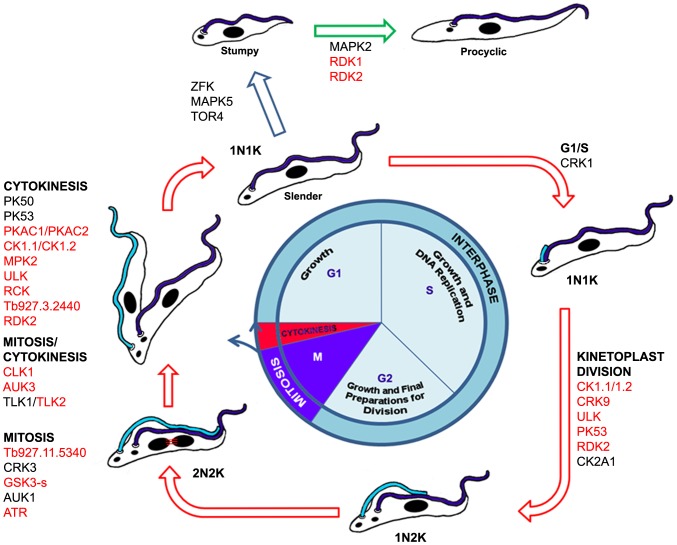
Schematic representation of the BSF trypanosome cell cycle and differentiation from BSF to PCF. Protein kinases implicated in cell cycle control and differentiation are indicated (red font: cell cycle function identified in this study; black font: cell cycle function previously identified).

Cell division in trypanosomes is tightly regulated and genetic perturbation leads readily to the formation of aberrant cells [10]. 22 PKs gave a defined cell cycle defect upon RNAi induction, with the majority blocked in mitosis or cytokinesis and surprisingly few in G1/S (Figure 6). A more defined cell cycle analysis that focuses on G1/S-specific markers could be designed to identify PKs involved in regulating G1 transition and entry into S phase, early cell cycle events such as basal body duplication or Golgi replication, DNA replication or positive regulators of differentiation that promote formation of cell cycle arrested stumpy form parasites. However, major challenges for the future will be to link individual PKs in signalling cascades, and, given that a high proportion of trypanosome PKs are differentially phosphorylated between the bloodstream and procyclic form ([19], [46] and Table S1), to determine life cycle stage-specific signalling pathways as well as identify those that are divergent in other eukaryotes.

The study also identified the first two PKs involved in repressing BSF to PCF differentiation. Although depletion of either RDK1 or RDK2 promotes BSF to PCF differentiation, key differences were noticed. Irrespective of the approaches used to induce differentiation of logarithmically growing *T. brucei* 2T1, a maximum of 60% of cells within the population were found to be receptive to signals of differentiation under the time frames studied here. However, only 20% of cells undergo differentiation upon RDK1 RNAi and these appear to be separate from the cells that are induced to differentiate when PTP1 is inhibited by BZ3. In contrast, 40–60% of cells undergo differentiation upon RDK2 RNAi suggesting that RDK2 might act to prevent differentiation of all cells within the population that are receptive to the various differentiation signals, and as such might act upstream of both RDK1 and PTP1. The identification of two protein kinases (this study) and two phosphatases [7], [8], which act as repressors of differentiation (Figure 6), highlight the importance of cell signalling by reversible phosphorylation in preventing inadvertent expression of procyclic-specific genes in the mammalian bloodstream. The phenotype induced by RDK1 and RDK2 (expression of procyclin) would be suitable for a target-based, phenotype-directed high content cell screen for regulators of differentiation using protein kinase focussed chemical libraries. Specific anti-RDK inhibitors would be useful chemical tools for investigating trypanosome differentiation and could also be potential lead compounds for drug development.

## Materials and Methods

### Ethics statement

All animal procedures were undertaken in adherence to experimental guidelines and procedures approved by The Home Office of the UK government. All work was covered by Home Office Project Licence PPL60/4442 entitled “Molecular Genetics of Trypanosomes and *Leishmania*”. All animal protocols received approval from the University of Glasgow Ethics Committee.

### Generation of kinome RNAi library

The pRPa^ISL^ plasmid [25] was redesigned *in silico* to contain two, inverted Gateway donor sites (containing attP sites and a *ccdB* counter selectable marker) separated by 150 bp of the *lacZ* gene. This modification was synthesised (Blue Heron Biotech) and cloned between the *Bam*HI and *Xba*I sites of pRPA^ISL^. The resultant plasmid, termed pGL2084, was propagated in *ccdB* Survivor cells (Invitrogen) at 25°C. RNAi target sequences were determined for the CDS of each PK gene using the TrypanoFAN: RNAit programme [47]. RNAi targets were amplified from *T. b. brucei* TREU 927 genomic DNA using Phusion high fidelity polymerase (NEB, Massachusetts) with appropriate oligonucleotide pairs (Table S3) incorporating attB1 and attB2 sites before being cloned into pGL2084 in a BP Recombinase reaction (Invitrogen) as per the manufacturer's instructions. 12 PKs were cloned into unmodified pRPa^ISL^ (indicated in Table S1). The resultant plasmids (pTL) were propagated using DH5α Max Efficiency cells, and purified and digested with *Asc*I (NEB) prior to transfection.

*T. b. brucei* 2T1 BSF cells and derivatives were maintained in HMI-11 (HMI-9 (GIBCO), 10% v/v foetal calf serum (FCS; GIBCO 10270), Pen/Strep (SIGMA) (penicillin 20 Uml^−1^, streptomycin 20 µgml^−1^)), at 37°C, 5% CO_2_ in vented flasks [48]. Appropriate selective drugs were added at the following concentrations: 2 µg ml^−1^ puromycin, 2.5 µg ml^−1^ phleomycin (InvivoGen), 5 µg ml^−1^ hygromycin B (Calbiochem) and 10 µg ml^−1^ blasticidin (InvivoGen). BSF parasites were transfected as previously described [27], with independent clones obtained by limiting dilution. Clones were screened for puromycin sensitivity [25]; puromycin sensitive clones were prioritised for further analyses.

In order to specifically target each individual *NEK12* mRNA, the *NEK12.1* and *NEK12.2* genes were aligned and short regions (over 20 nt) were identified where enough divergence existed to enable gene specific silencing by RNAi. For each gene, five small fragments (targeting the equivalent regions of each gene) were identified and joined *in silico*, then synthesised into attB flanked inserts (Genscript) ready for Gateway cloning into pGL2084. CLK1/2 individual RNAi constructs were generated by targeting the diverged 5′ region of the CDS and 5′ UTR.

### Assessment of growth and cell cycle status following RNAi induction

Cell cultures were adjusted to 2×10^4^ cells ml^−1^ and divided into two pools. RNAi was induced in one pool by the addition of tetracycline to a final concentration of 1 µg ml^−1^ in 70% ethanol, while the other was treated with an equivalent volume of 70% ethanol. 200 µl of each culture was then plated in triplicate into wells of a 96 well plate and incubated for 48 hr at 37°C in the presence of 5% CO_2_. Twenty microlitres of Alamar Blue (0.49 mM resazurin in phosphate-buffered saline (PBS)) was added to each well and the plates incubated for a further 24 hr. The plate was then read at λ_excitation_ 485 nm and λ_emission_ 620 nm in an Envision Plate Reader (Perkin Elmer). The average ratio of the values for induced wells to uninduced wells was then calculated. A value of 1 indicated that RNAi induction did not affect cell proliferation, while a value <1 indicated RNAi induction reduced cell proliferation to some extent. A cut-off value of <0.9 was used in this study to determine which genes were analysed further.

To confirm the RNAi growth defects detected in the Alamar Blue assay for cell lines with values <0.9, one clone of each cell line was induced with 1 µg ml^−1^ tetracycline. Cell densities were determined every 24 hr and cumulative growth curves plotted taking into account the dilution factors necessary to maintain cultures below a density of 1×10^6^ cells ml^−1^. For analysis of growth for RDK1 and RDK2 RNAi, 30 ml of HMI-11 was seeded with10^5^ cells ml^−1^ and divided into 5 ml aliquots in a 6 well plate. Three wells were induced with 1 µg ml^−1^ tetracycline, and cumulative growth curves generated as described above.

Mouse infections were carried out in ICR mice; initially, a donor mouse was infected with 5×10^4^ cultured parasites and monitored for 72 hr. Experimental groups of 4 mice were infected using 1×10^5^ parasites taken from the same donor mouse. Doxycycline was added to drinking water after 48 hr to induce gene specific RNAi in the infecting trypanosomes. Parasitaemia was monitored daily by haemocytometer cell counting of blood (diluted in 0.83% ammonium chloride) taken by tail venipuncture. The cell cycle profile of RNAi cell lines over time following induction was determined by staining cells with DAPI and cataloguing the nucleus/kinetoplast (N/K) configuration of >200 intact cells/timepoint [35]. For *CLK1* RNAi, the cytokinesis stage of >200 2N2K cells/timepoint was also determined as previously described [49]. Flow cytometry was carried out as described previously [35] but using FlowJo version 7.6.5 for analysis (Tree Star).

### RNA analysis

For qPCR analysis of RNAi lines, 2×10^7^ cells were harvested from induced and uninduced cultures at 24 hr post-induction and total RNA was isolated using the Qiagen RNeasy Kit (with on column DNaseI digest) using a Qiacube Robot to minimize RNase contamination. Samples were then further treated with RQ1 RNase-free DNaseI (Promega). To prepare cDNA, 2 µg of total RNA were used as a template in a reverse transcriptase reaction with random hexamers using the SuperScript Reverse Transcriptase III system (Invitrogen). Following cDNA synthesis, *E. coli* RNase H (Invitrogen) was added to degrade the template RNA. qPCR reactions were set up using Applied Biosystems SYBR Green PCR master mix as described previously [27]. A denaturation step was added to determine that only a single product was formed by each primer pair; primer pairs were also tested for efficiency against the control primers to ensure accurate comparisons could be made. The control primers used targeted the C1 gene (Tb927.10.12970) due to its stability during trypanosome life cycle differentiation [20]. Assays were also performed using gene specific primers in a One STEP SYBR PrimeScript Kit II (Takara), to increase throughput; this technique was validated against samples analysed by the Monnerat *et al.*, method [27].

For RNAseq analysis, total RNA from *RDK1* RNAi cell lines (2 biological replicates, clone 1 and clone 2) was extracted from induced (48 hr) and uninduced cultures, then prepared for RNAseq at Glasgow Polyomics Facility (www.gla.ac.uk/polyomics). mRNA was amplified using oligo-dT and multiplexed, before being sequenced using an Illumina Genome Analyzer IIx. This generated paired-end reads (4 million per sample, with an average size of 110 bases), with an average insert size of 200 bases. The read quality was controlled by trimming on mean quality score >Phred 20 (equating to <1% error rate). Non-redundant read data were aligned to the *T. b. brucei* 927 reference genome from TriTrypDB version 4.0 using Bowtie (0.12.7), and analysed for differential gene expression using Cufflinks (1.3.0). Cufflinks determined differentially regulated genes by calculating an uncorrected p-value for the fold change in reads for a gene and a q-value (a corrected p-value using a false discovery rate of 0.05). If the p-value was less than the q-value, the gene was considered to be significantly differentially regulated. Expression values are reported as fragments per kilobase of exon per million reads mapped (FPKM).

### Subcellular fractionation and immunofluorescence

An allele of *RDK1* was tagged endogenously with a C-terminal 12Myc epitope using the pNAT BSD×12Myc vector [25]. Crude cell fractionation was performed using detergent based or hypotonic buffers as previously described [38]; Western blots of the lysates were probed with the mouse monoclonal anti-Myc antibody clone 9E10 (Millipore) to detect RDK1::12Myc, anti-oligopeptidase B (OPB) antibody (cytoplasmic control, [50]), the anti-β-tubulin antibody KMX (cytoskeleton control) [38]) and anti-metacaspase 4 (MCA4) (membrane protein control, [38]).

For immunofluorescence, 1×10^6^ cells were washed in Trypanosome Dilution Buffer (TDB; 20 mM Na_2_HPO_4_, 2 mM NaH_2_PO_4_, 80 mM NaCl, 5 mM KCl, 1 mM MgSO_4_, 20 mM glucose, pH 7.4) and fixed in 3% paraformaldehyde overnight at 4°C. After washing in PBS, cells were resuspended in 0.5 ml 0.1M Na_2_HPO_4_/0.1M glycine pH 7.2 and incubated on ice for 15 minutes. 0.5 ml PBS/0.2% Triton X-100 was added and cells were incubated for a further 5 minutes on ice before being washed in 0.5 ml PBS/1% bovine serum albumin (BSA) and resuspended in 100 µl 1/500 dilution of anti-Myc monoclonal antibody clone 4A6 (Millipore). Following a 1 hr incubation on ice, cells were washed twice in PBS/1% BSA and then incubated in a 1/100 dilution of AlexaFluor488-conjugated anti-mouse IgG (Invitrogen) for a further hr on ice in the dark. Cells were washed once in PBS/BSA and once in 100 mM HEPES pH 7.5 before being incubated in 10 µg ml^−1^ 4′,6-diamidino-2-phenylindole (DAPI)/100 mM HEPES pH 7.5 for 5 mins, washed and resuspended in 100 mM 4-(2-hydroxyethyl)piperazine-1-ethanesulfonic acid (HEPES) pH 7.5. Cells were examined on a DeltaVision RT epifluorescence imaging system with images taken with a Cool Snap HQ2 camera, deconvolved and processed using SoftWoRx software.

### Monitoring BSF to PCF differentiation

The differentiation of BSF to PCF trypanosomes was routinely monitored by flow cytometry as previously described [1]. Briefly, 3×10^6^ cells were washed in TDB, before being fixed in 2% formaldehyde v/v, 0.05% glutaraldehyde v/v at 4°C for 1 hr. After washing in PBS, the cells were labelled with 1/100 dilution of FITC-conjugated anti-EP procyclin mouse monoclonal IgG1 (CLP001F, clone TBRP1/247, Cedarlane Laboratories, Ontario) in PBS for 1 hr at room temperature. Cell fluorescence and scattering parameters were measured on a FACS-Calibur Flow Cytometer (Becton Dickinson), with 50 000 events being captured per sample. Data analysis was performed using FlowJo software (Tree Star Inc.) where gates were applied in the FSC/SSC channels to exclude cellular debris or aggregates before a bifurcation gate was applied to define EP procyclin positive/negative cells. *RDK1* or *RDK2* RNAi was induced for 0, 24 or 48 h prior to the addition of 250 µM 8-pCPT-cAMP [1] or 150 µM BZ3 (3-(3,5-Dibromo-4-hydroxy-benzoyl)-2-ethyl-benzofuran-6-sulfonicacid-(4-(thiazol-2-ylsulfamyl)-phenyl)-amide), (Merck), or prior to subjection to cold shock (27°C) for the final 24 hr of each experiment [7].

## Supporting Information

Figure S1 **The stem-loop RNAi construct cloning strategy.** The tetracycline-inducible, stem-loop RNAi vector, pRPa^ISL^ [25], [52], was modified in a similar way to the plant pHELLSGATE recombineering system [53]. A: Illustration of the main features of the RNAi plasmid. Grey *hyg*Δstop and *RRNA* boxes: sequences required for integration into the *T. b. brucei* 2T1 cell line; ald: aldolase 3′UTR; attP1 and attP2: Gateway cloning recombination sites; *ccdB*: negative selectable marker. Restriction endonuclease sites used to confirm correct integration are shown at the top of the figure. After transfection, the expression of the complete *HYG* gene is controlled by the EP procyclin promoter (*P_EP_*) and the RNAi sequence by the ribosomal RNA promoter (*P_RRNA_*), which has been modified by the inclusion of a Tet operator sequence to render it inducible by tetracycline. The position of a 150 bp *LacZ* stuffer fragment, which allows *E. coli* to replicate the plasmid backbone without an insert, is indicated. B: depiction of the recombination of a single PCR product into the vector in two opposing, reverse complemented orientations via the Gateway system BP clonase reaction. Recombination of the attB1 and attB2 sites flanking the PCR product with the attP1 and attP2 that flank the *ccdB* gene of the vector generates attL1 and attL2 sites and results in the excision of the ccdB marker.
(PDF)

Figure S2 **Cell cycle analysis.** RNAi cell lines exhibiting a loss of fitness following induction by Alamar Blue assay were further screened for cell cycle defects. Growth curves were performed for 96 hours following RNAi induction with tetracycline (Tet) and DAPI staining was performed to determine the nuclei/kinetoplast (N/K) configurations of cells over time (*n*>200 cells/timepoint). ‘Other’ cells are those with abnormal N/K configurations, details of which can be viewed in Table S2. A: Cumulative growth curves for RNAi cell lines exhibiting no growth defect following induction; B: RNAi cell lines displaying slow growth phenotype, but no cell cycle defect following induction; C: RNAi cell lines displaying slow growth accompanied by a cell cycle defect following induction; D: RNAi cell lines displaying growth arrest phenotype, but no cell cycle defect following induction; E: RNAi cell lines displaying a growth arrest and a cell cycle defect following induction; F: RNAi cell lines displaying a cell death phenotype, but no cell cycle defect following induction; G: RNAi cell lines displaying cell death and a cell cycle defect following induction. H: Flow cytometry analysis for selected clones. The DNA content of each peak is indicated.
(PDF)

Figure S3 **Quantification of mRNA knockdown in selected RNAi cell lines.** A: qRT-PCR was performed to assess mRNA levels (corresponding to the targeted gene) in induced (+Tet) and uninduced (−Tet) RNAi cell lines at 24 hr post-induction. Mean relative quantification values from 4 technical replicates with their standard deviations are presented. Unpaired t-tests were performed for each set of data, with asterisks indicating significant differences (p<0.05). Red bars indicate genes for which a LOF phenotype was detected while grey bars indicate a gene for which no LOF phenotype was observed. B: qRT-PCR results for dual and individual NEK12 RNAi cell lines to assess specificity of RNA knockdown.
(PDF)

Figure S4
**RNAi of NEK12.1/12.2.** A: Cumulative cell counts over time following tetracycline (Tet) induction (+) or not (−) of *NEK 12.1/NEK12.2* dual RNAi cell line grown in culture. Cell densities were maintained between 10^5^ and 10^6^ cells ml^−1^. Error bars indicate the standard deviations around the means of three technical replicates. Inset: Analysis of NEK12.1 protein knockdown following RNAi induction. *NEK12.1/NEK12.2* RNAi cell line clone 1 expressing *GFP-TY::NEK12.1* from the endogenous locus was analysed by Western blotting with an anti-GFP antibody 24 hr after RNAi induction (+T) or not (−T). anti-EF1α antibody was used as a loading control. B: Proliferation of *NEK12.1/NEK12.2* RNAi line in mice. 1×10^5^ trypanosomes were inoculated in 4 mice and RNAi induced with doxycycline (Dox, as indicated) in 2 mice 24 hr later. Uninduced mice were culled as indicated (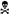) when their parasitaemias rose above 10^8^ cells ml^−1^.(PDF)

Table S1 **Summary of the characteristics of the RNAi cell lines.** Alsford RITseq RNAi screen [24] loss of fitness (LOF) phenotypes are highlighted orange (Day 3) or red (Day 6). Mackey [21], Merritt [23] and Nishino [22] screens LOF phenotypes are highlighted red. For the screen described in this study, Alamar blue ratios <0.9 are highlighted in brown. Asterisks indicate RNAi cell lines targeting ≥1 PK. Phosphorylation status of PKs is taken from [46].
(XLSX)

Table S2 **Details of abnormal cells accumulating following RNAi induction as observed by DAPI staining.** Actual numbers and percentages of each cell type observed are shown. Separate tabs for each PK are provided and, for ease of reference, are colour coded according to the RNAi phenotype (as assigned in Figure S2A–G). Orange: no growth defect following induction; purple: RNAi cell lines displaying slow growth phenotype, but no cell cycle defect following induction; lime: RNAi cell lines displaying slow growth accompanied by a cell cycle defect following induction; grey: RNAi cell lines displaying growth arrest phenotype, but no cell cycle defect following induction; pink: RNAi cell lines displaying a growth arrest and a cell cycle defect following induction; blue: RNAi cell lines displaying a cell death phenotype, but no cell cycle defect following induction; yellow: RNAi cell lines displaying cell death and a cell cycle defect following induction.
(XLSX)

Table S3 **List of differentially expressed genes identified by RNAseq following induction of RDK1 RNAi for 48 hr.**
(XLSX)

Table S4 **Oligonucleotides used in this study.** Separate tabs give details of (**A**) primer sequences used for constructing pre-Gateway RNAi plasmids along with (**B**) a list of these plasmids, (**C**) primer sequences for constructing Gateway RNAi plasmids, (**D**) the RNAi inserts for the NEK12-specific RNAi constructs, (**E**) qRT-PCR primers and (**F**) primers used to clone the RDK1 endogenous tagging construct.
(XLSX)
