# Supplementary figures and images for: Regulators of Trypanosoma brucei Cell Cycle Progression and Differentiation Identified Using a Kinome-Wide RNAi Screen
Source: PLoS Pathog. 2014 Jan 16;10(1):e1003886. doi: 10.1371/journal.ppat.1003886 (PMC3894213; doi:10.1371/journal.ppat.1003886)

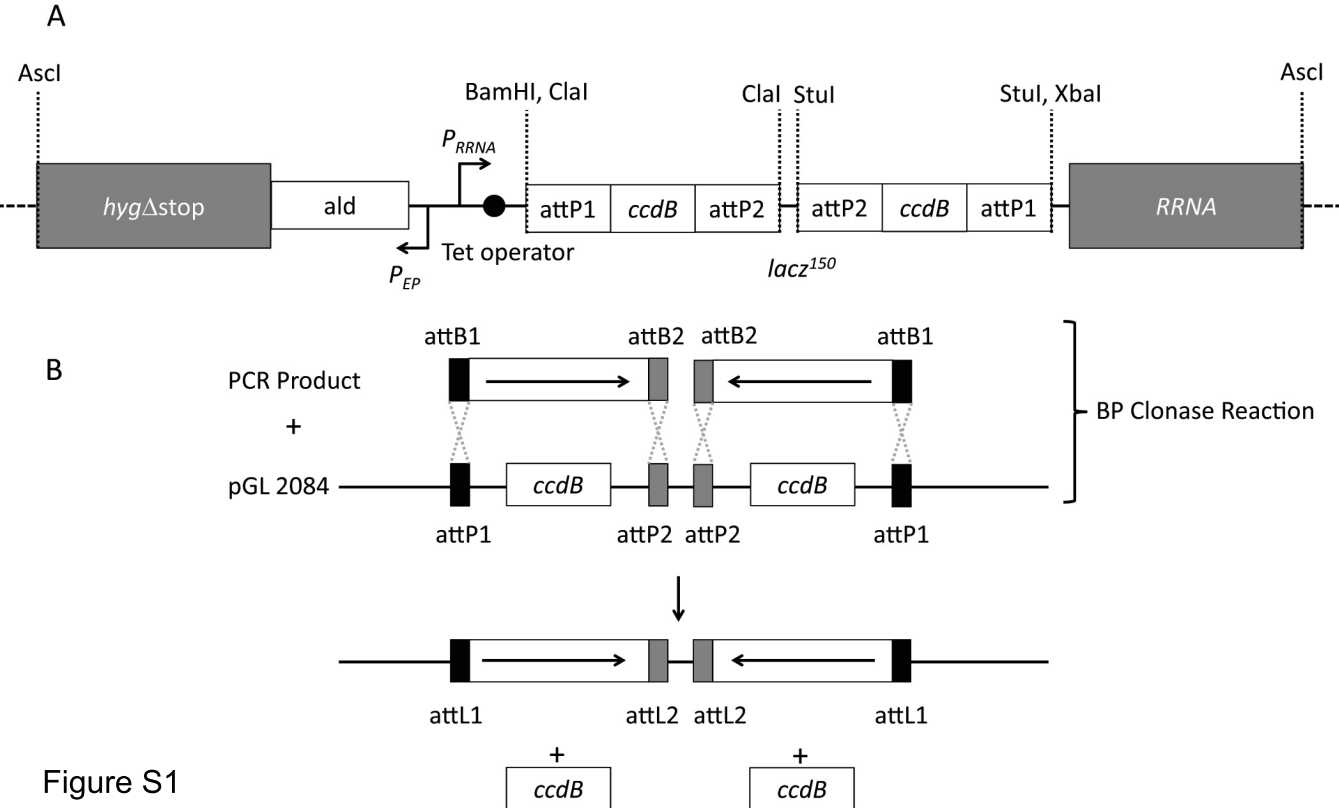

Figure S1

Supplement: Figure S1 — The stem-loop RNAi construct cloning strategy. The tetracycline-inducible, stem-loop RNAi vector, pRPaISL [25], [52], was modified in a similar way to the plant pHELLSGATE recombineering system [53]. A: Illustration of the main features of the RNAi plasmid. Grey hygΔstop and RRNA boxes: sequences required for integration into the T. b. brucei 2T1 cell line; ald: aldolase 3′UTR; attP1 and attP2: Gateway cloning recombination sites; ccdB: negative selectable marker. Restriction endonuclease sites used to confirm correct integration are shown at the top of the figure. After transfection, the expression of the complete HYG gene is controlled by the EP procyclin promoter (PEP) and the RNAi sequence by the ribosomal RNA promoter (PRRNA), which has been modified by the inclusion of a Tet operator sequence to render it inducible by tetracycline. The position of a 150 bp LacZ stuffer fragment, which allows E. coli to replicate the plasmid backbone without an insert, is indicated. B: depiction of the recombination of a single PCR product into the vector in two opposing, reverse complemented orientations via the Gateway system BP clonase reaction. Recombination of the attB1 and attB2 sites flanking the PCR product with the attP1 and attP2 that flank the ccdB gene of the vector generates attL1 and attL2 sites and results in the excision of the ccdB marker. (PDF) [file ppat.1003886.s001.pdf]

A

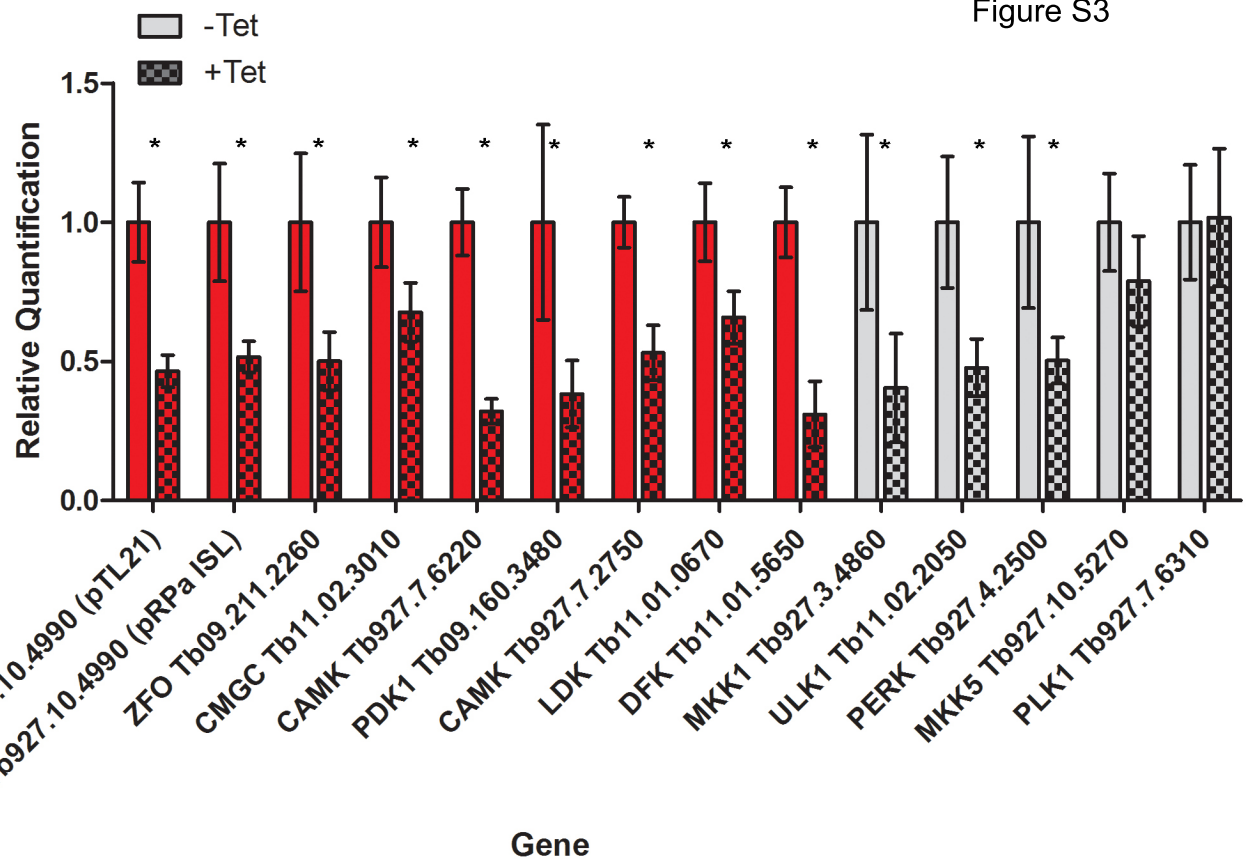

B

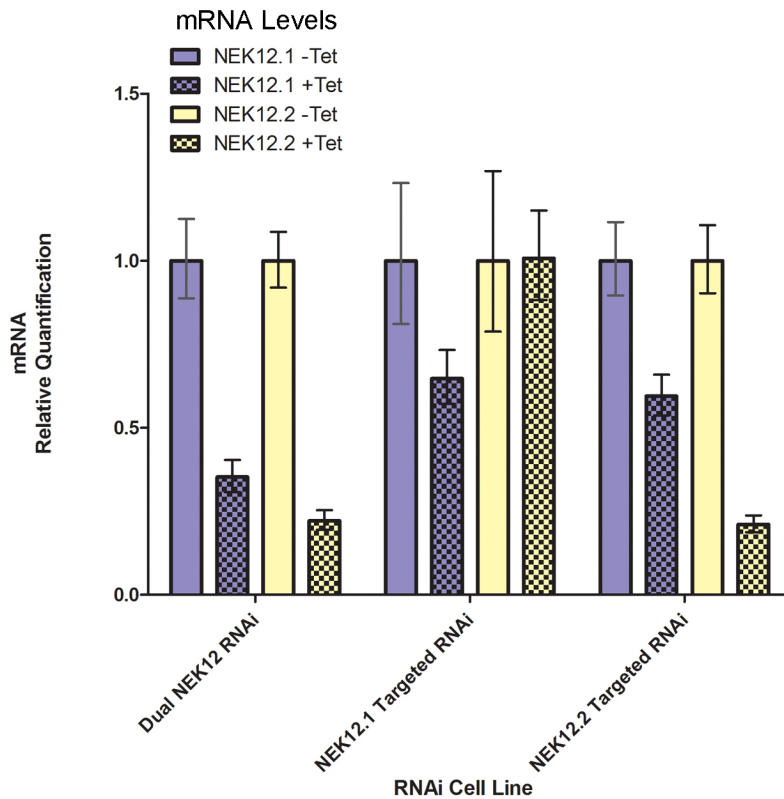

Supplement: Figure S3 — Quantification of mRNA knockdown in selected RNAi cell lines. A: qRT-PCR was performed to assess mRNA levels (corresponding to the targeted gene) in induced (+Tet) and uninduced (−Tet) RNAi cell lines at 24 hr post-induction. Mean relative quantification values from 4 technical replicates with their standard deviations are presented. Unpaired t-tests were performed for each set of data, with asterisks indicating significant differences (p<0.05). Red bars indicate genes for which a LOF phenotype was detected while grey bars indicate a gene for which no LOF phenotype was observed. B: qRT-PCR results for dual and individual NEK12 RNAi cell lines to assess specificity of RNA knockdown. (PDF) [file ppat.1003886.s003.pdf]

Figure S4

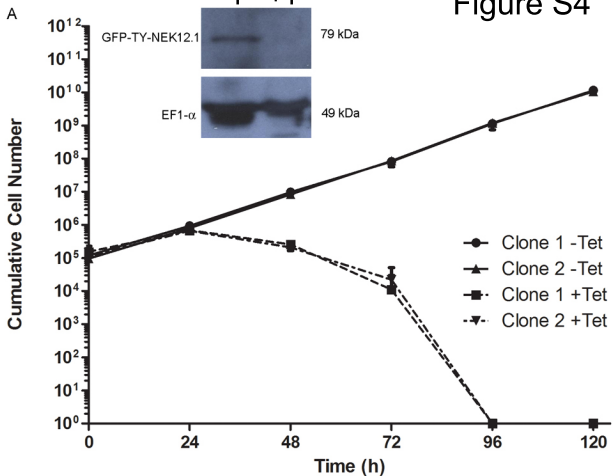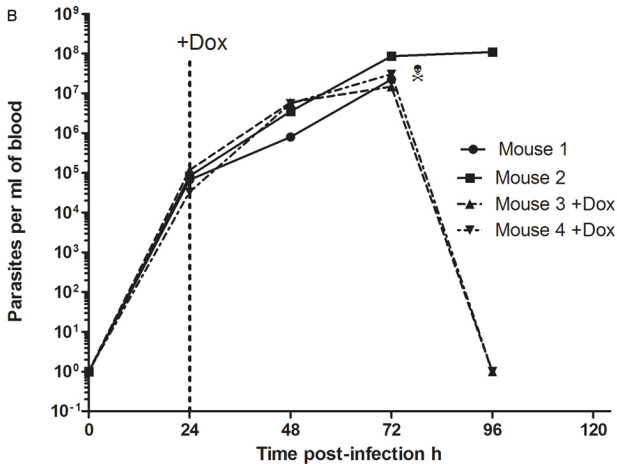

Supplement: Figure S4 — RNAi of NEK12.1/12.2. A: Cumulative cell counts over time following tetracycline (Tet) induction (+) or not (−) of NEK 12.1/NEK12.2 dual RNAi cell line grown in culture. Cell densities were maintained between 105 and 106 cells ml−1. Error bars indicate the standard deviations around the means of three technical replicates. Inset: Analysis of NEK12.1 protein knockdown following RNAi induction. NEK12.1/NEK12.2 RNAi cell line clone 1 expressing GFP-TY::NEK12.1 from the endogenous locus was analysed by Western blotting with an anti-GFP antibody 24 hr after RNAi induction (+T) or not (−T). anti-EF1α antibody was used as a loading control. B: Proliferation of NEK12.1/NEK12.2 RNAi line in mice. 1×10^5^ trypanosomes were inoculated in 4 mice and RNAi induced with doxycycline (Dox, as indicated) in 2 mice 24 hr later. Uninduced mice were culled as indicated () when their parasitaemias rose above 108 cells ml−1. (PDF) [file ppat.1003886.s004.pdf]
