# Supplementary material for: Regulators of Trypanosoma brucei Cell Cycle Progression and Differentiation Identified Using a Kinome-Wide RNAi Screen
Source: PLoS Pathog. 2014 Jan 16;10(1):e1003886. doi: 10.1371/journal.ppat.1003886 (PMC3894213; doi:10.1371/journal.ppat.1003886)
Supplement: Figure S2 — Cell cycle analysis. RNAi cell lines exhibiting a loss of fitness following induction by Alamar Blue assay were further screened for cell cycle defects. Growth curves were performed for 96 hours following RNAi induction with tetracycline (Tet) and DAPI staining was performed to determine the nuclei/kinetoplast (N/K) configurations of cells over time (n>200 cells/timepoint). ‘Other’ cells are those with abnormal N/K configurations, details of which can be viewed in Table S2. A: Cumulative growth curves for RNAi cell lines exhibiting no growth defect following induction; B: RNAi cell lines displaying slow growth phenotype, but no cell cycle defect following induction; C: RNAi cell lines displaying slow growth accompanied by a cell cycle defect following induction; D: RNAi cell lines displaying growth arrest phenotype, but no cell cycle defect following induction; E: RNAi cell lines displaying a growth arrest and a cell cycle defect following induction; F: RNAi cell lines displaying a cell death phenotype, but no cell cycle defect following induction; G: RNAi cell lines displaying cell death and a cell cycle defect following induction. H: Flow cytometry analysis for selected clones. The DNA content of each peak is indicated. (PDF) [file ppat.1003886.s002.pdf]

Figure S2

A

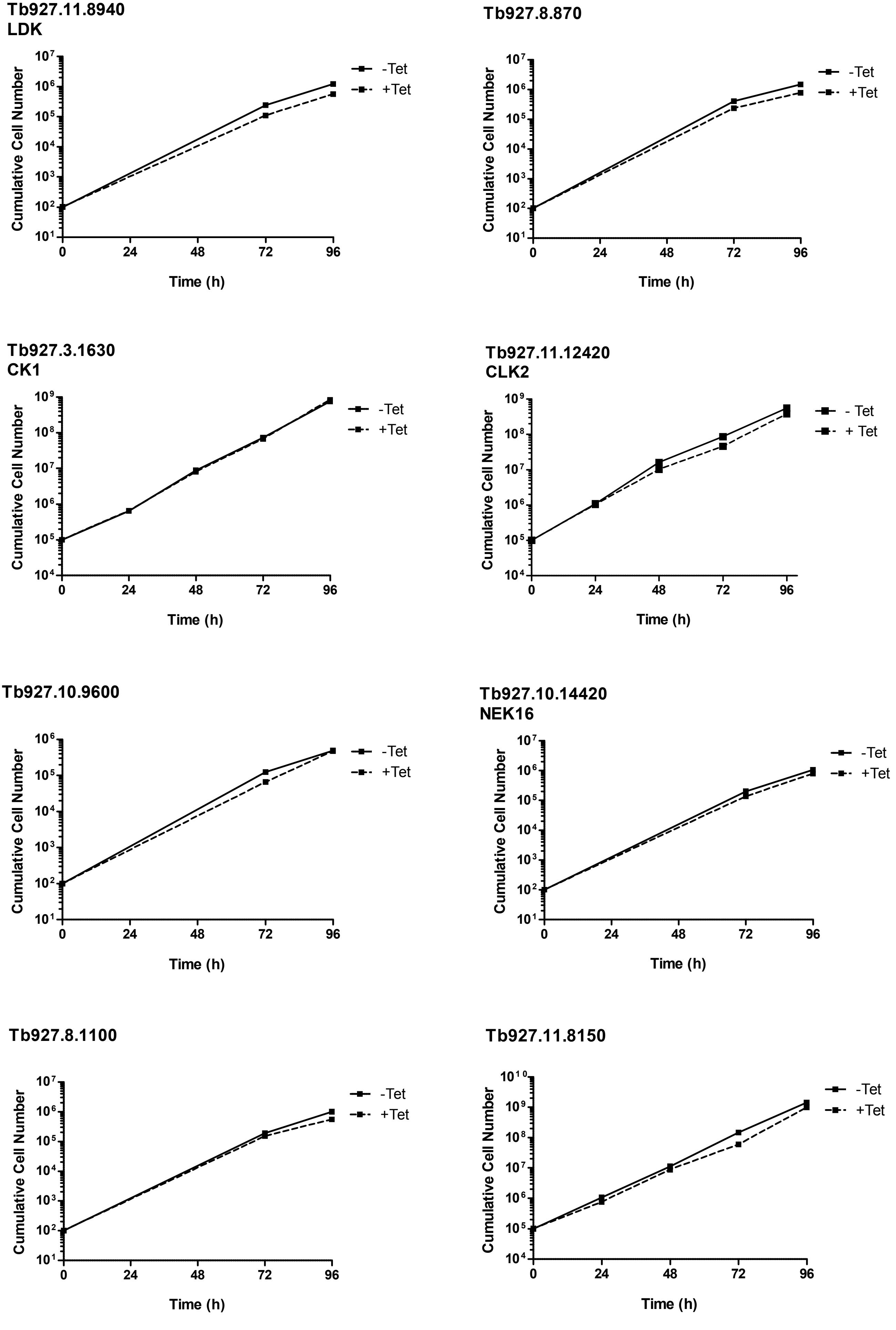

Tb927.11.8940
LDK
Tb927.8.870
Tb927.3.1630
CK1
Tb927.11.12420
CLK2
Tb927.10.9600
Tb927.10.14420
NEK16
Tb927.8.1100
Tb927.11.8150
Cumulative Cell Number
Time (h)
-Tet
+Tet
- Tet
+ Tet
0
24
48
72
96

Tb927.10.9900

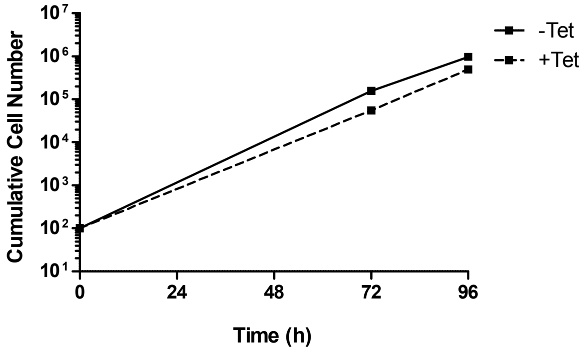

B

Tb927.9.4910
PDK1

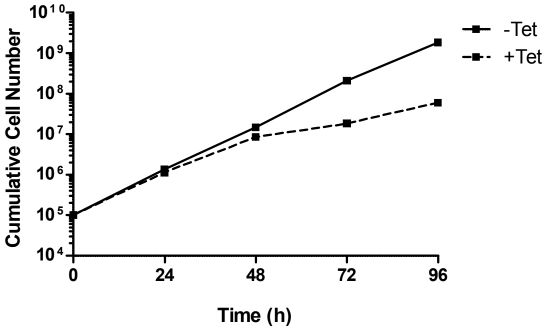

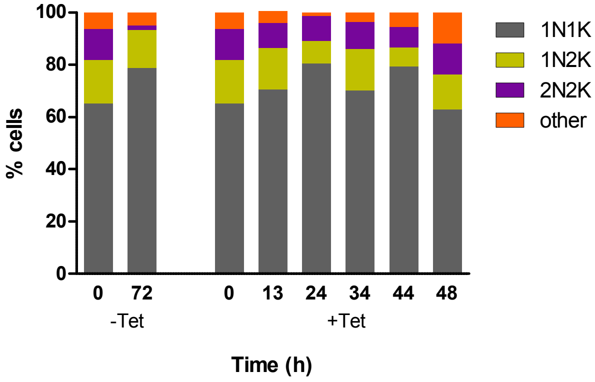

Tb927.7.2750

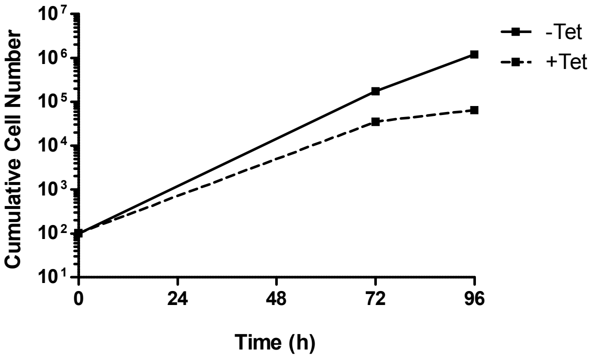

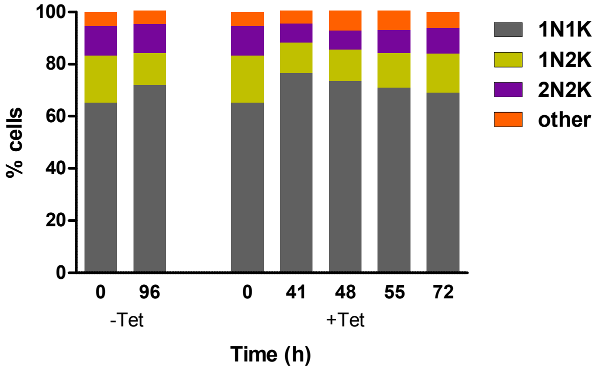

Tb927.7.7360
CRK2

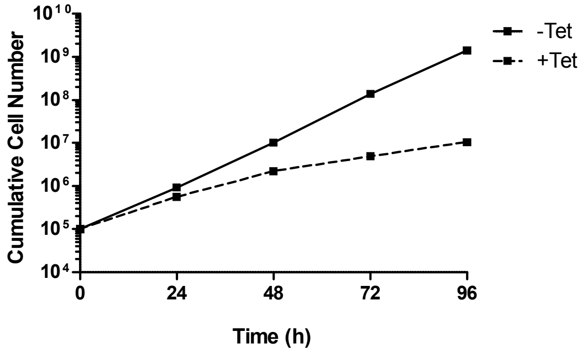

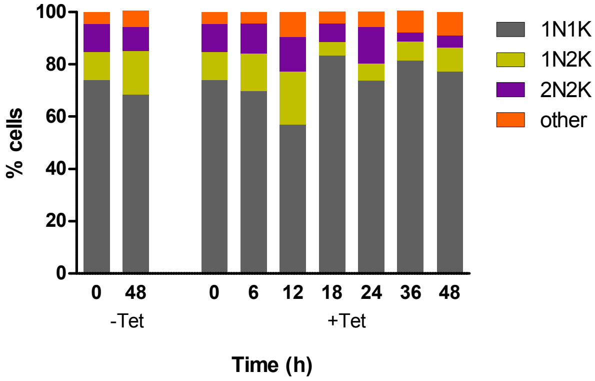

Tb927.11.1180
CRK6

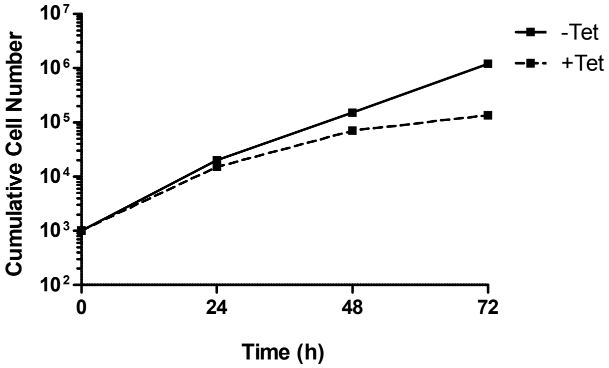

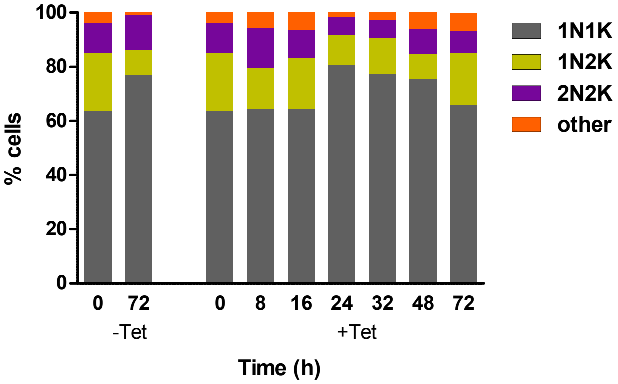

Tb927.10.7780
KFR1

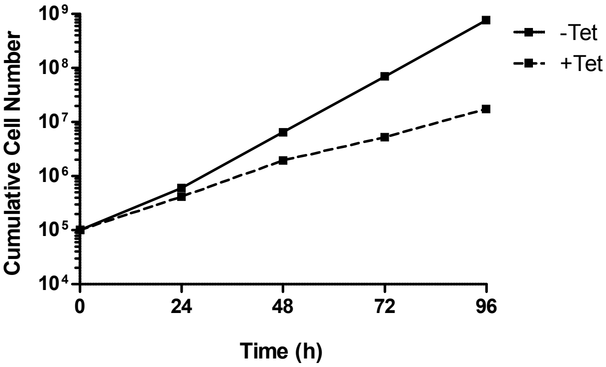

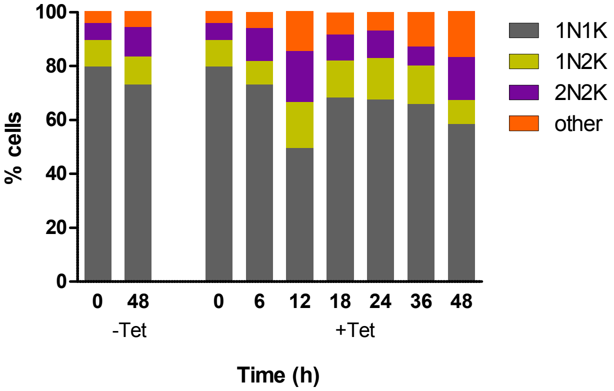

Tb927.10.15300

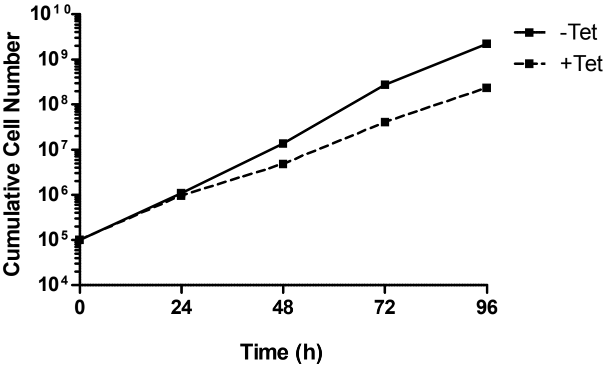

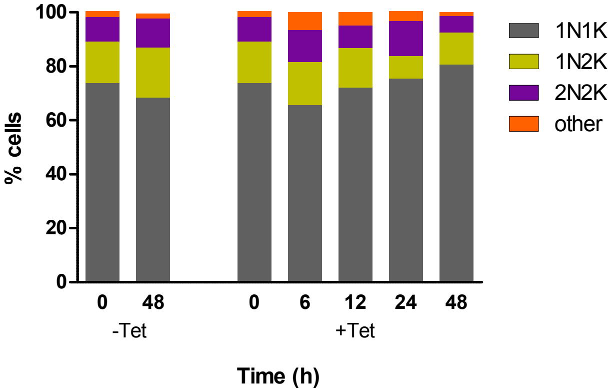

Tb927.4.3420
WEE1

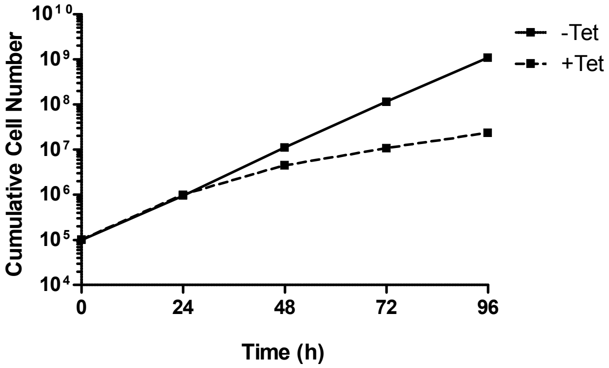

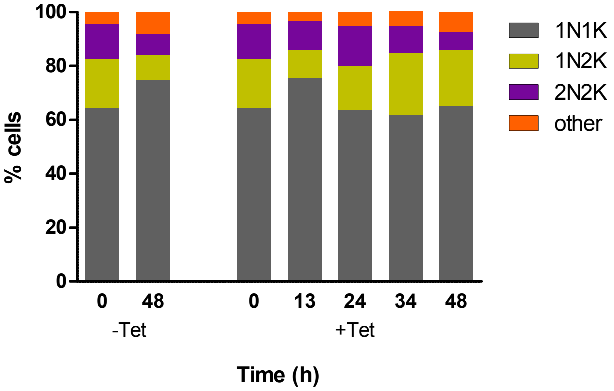

Tb927.11.14070
RDK1

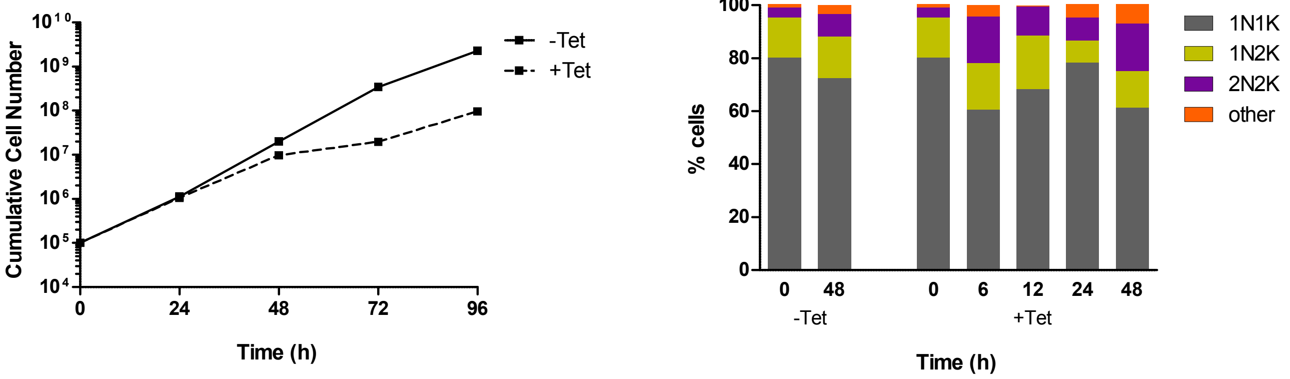

C

Tb927.7.5770
PK53

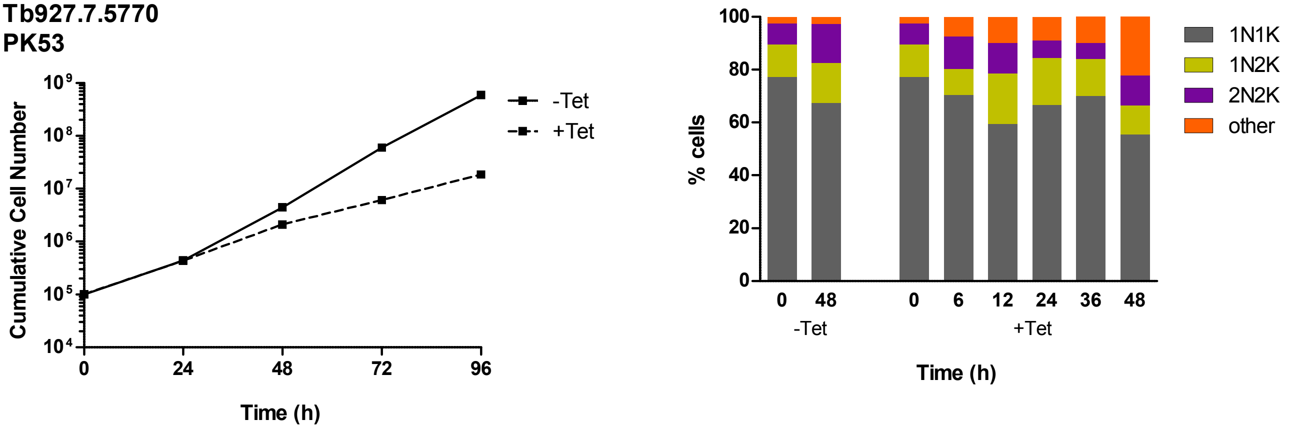

Tb927.9.11100&Tb927.9.11030
PKAC1&PKAC2

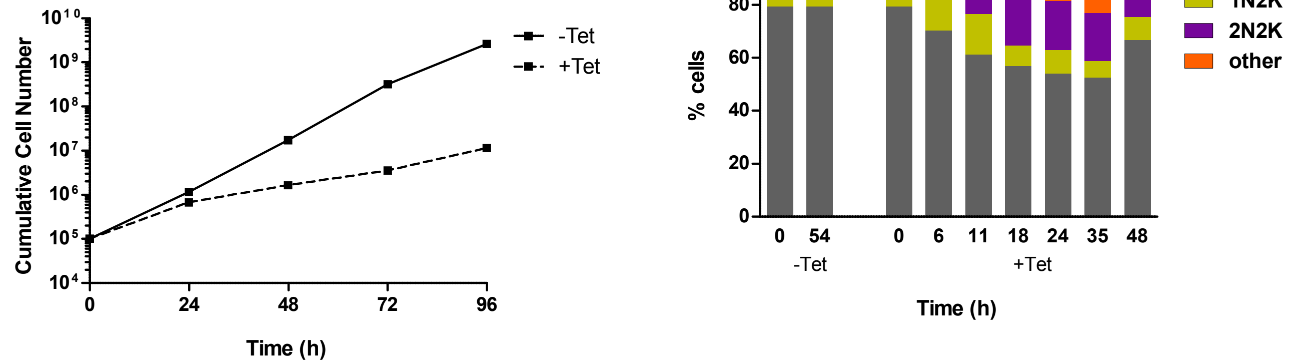

Tb927.11.5340

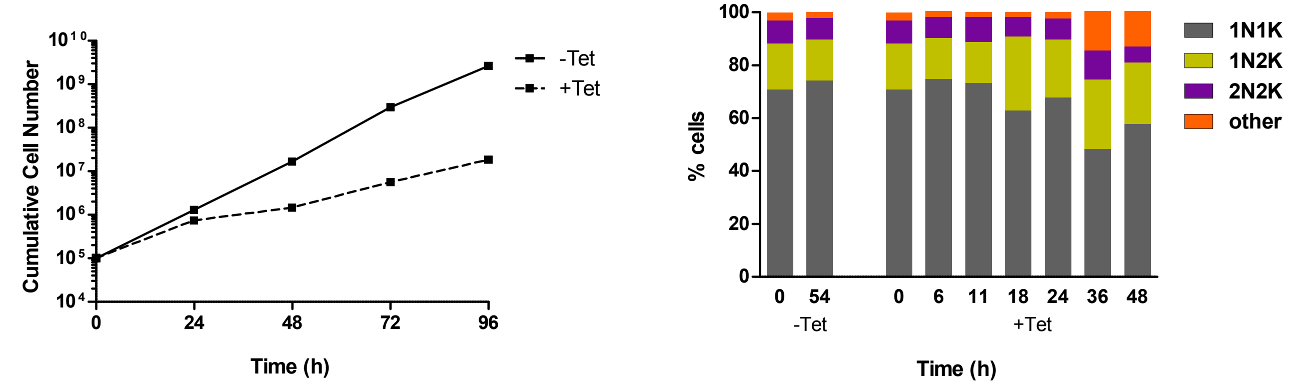

Tb927.10.1070
CRK1

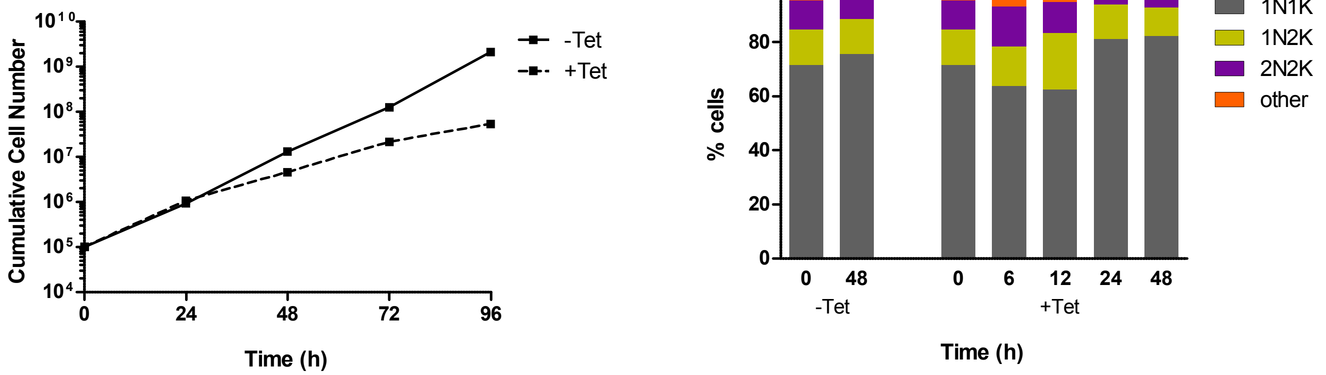

Tb927.9.1670
AUK3

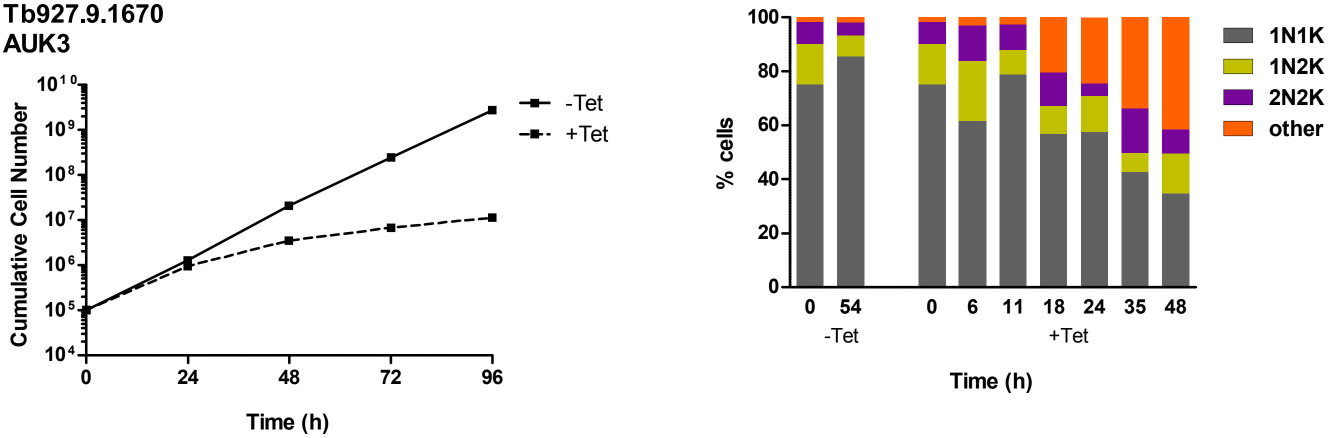

Tb927.7.3210

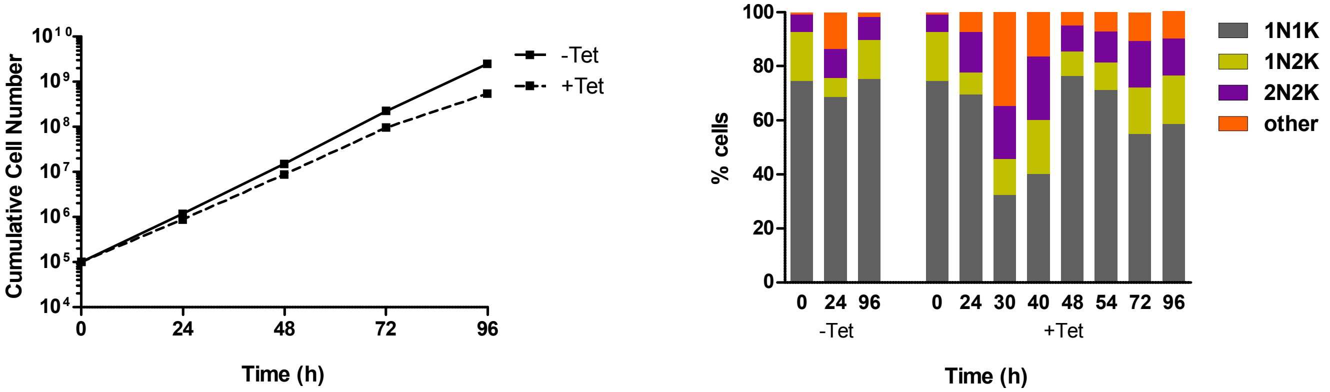

Tb927.10.8420
TOR1

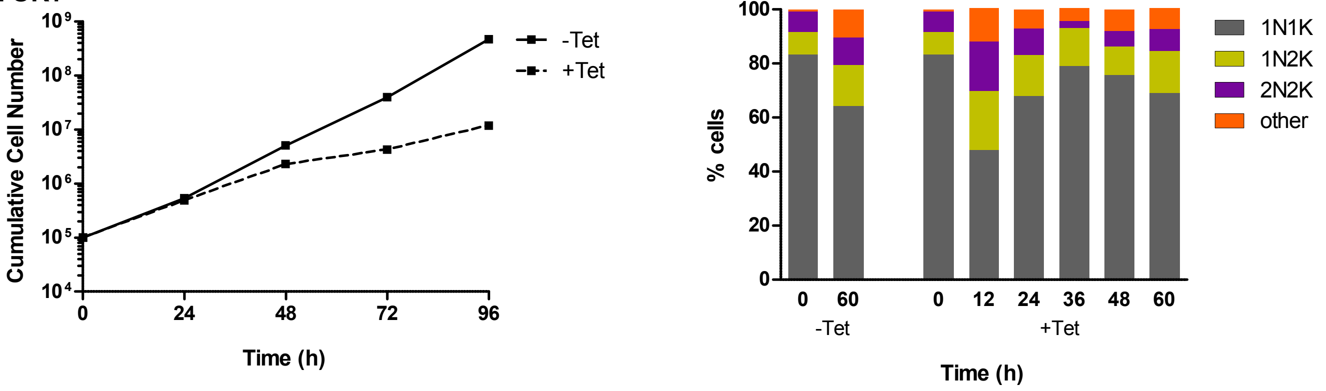

Tb927.1.1930
TOR4

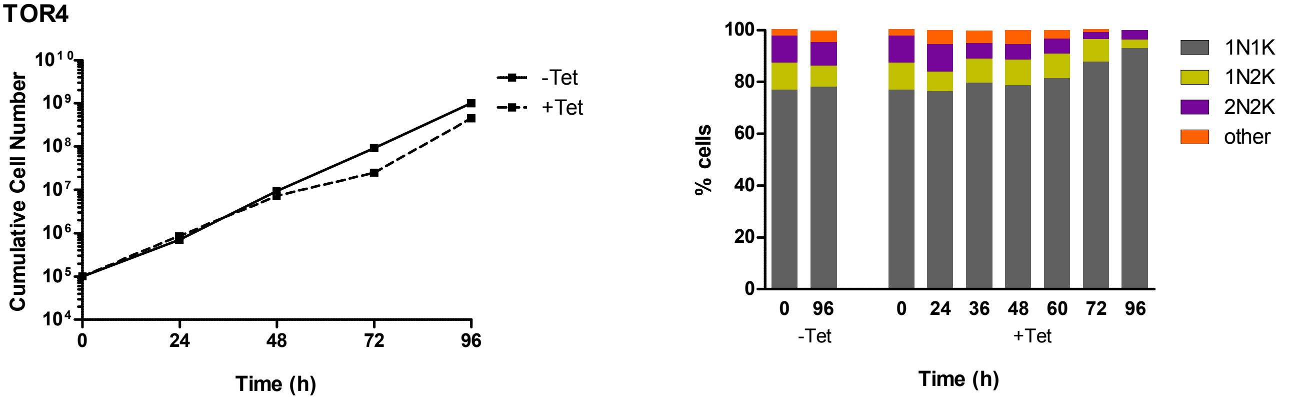

D

Tb927.6.4970

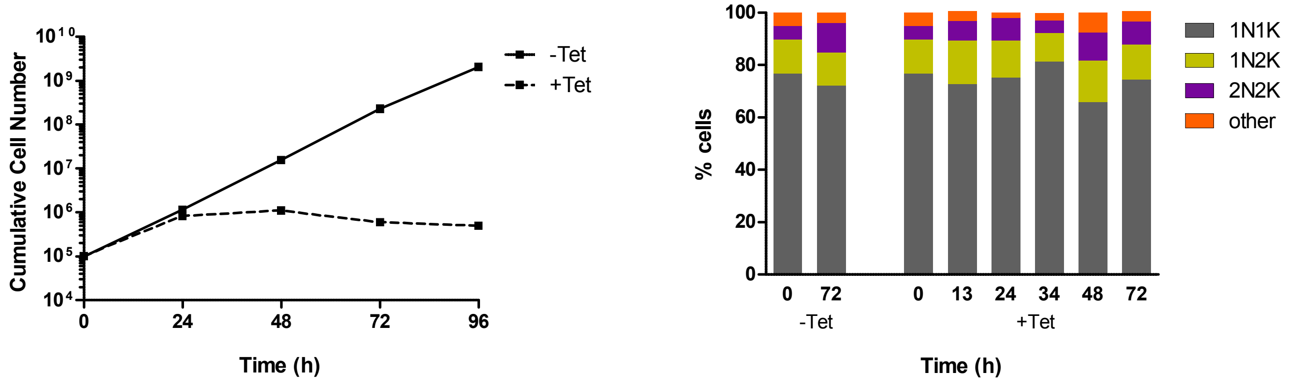

Tb927.9.10920
PK6

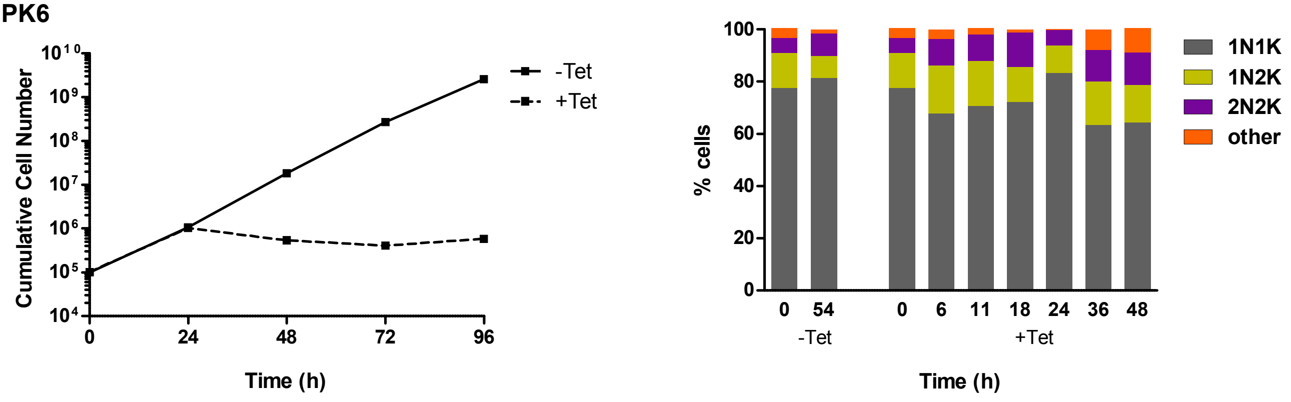

Tb927.11.2040

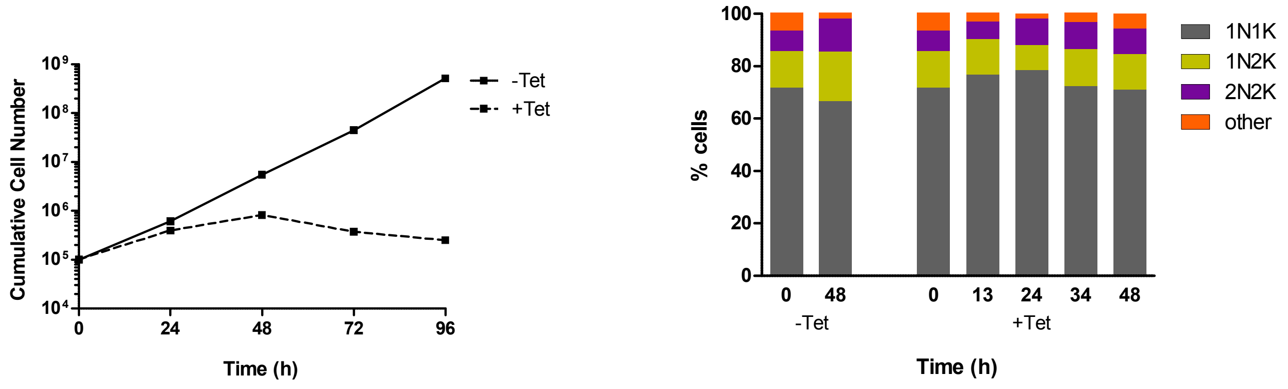

Tb927.8.5730
SLK1

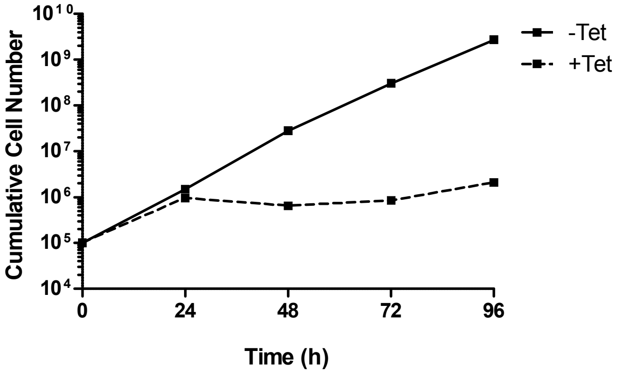

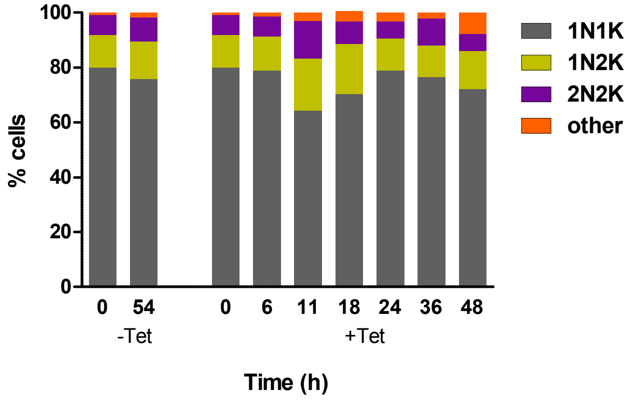

Tb927.9.12880
SLK2

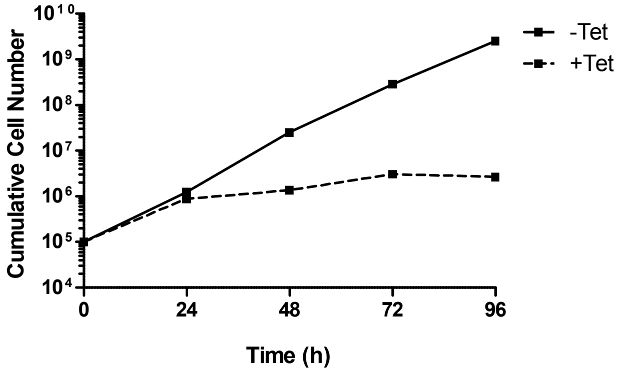

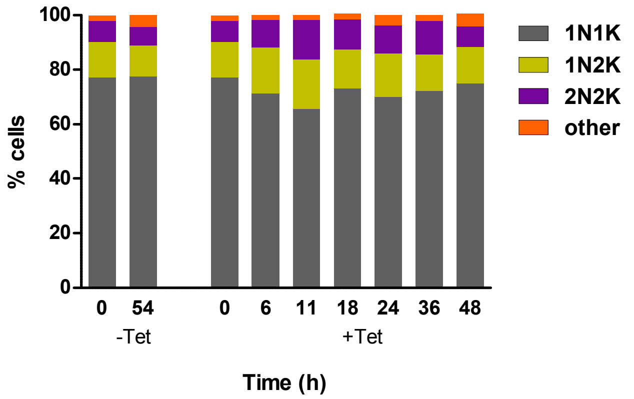

Tb927.9.6560

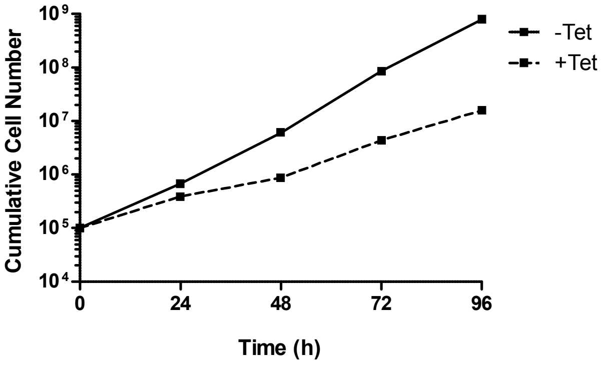

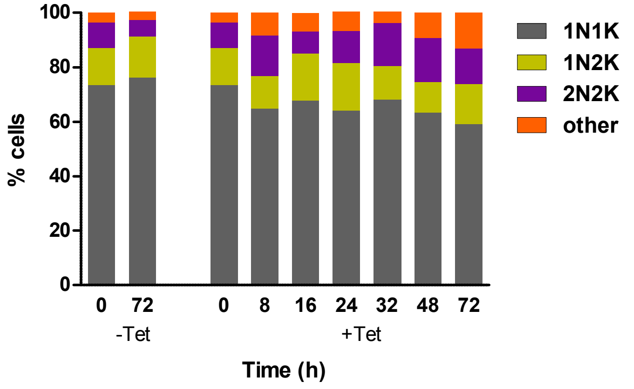

Tb927.3.5400
RIO1

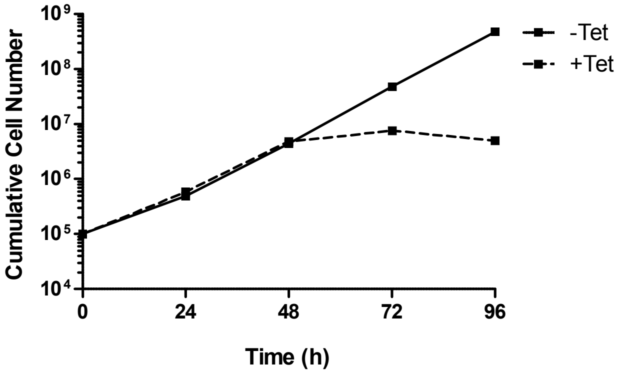

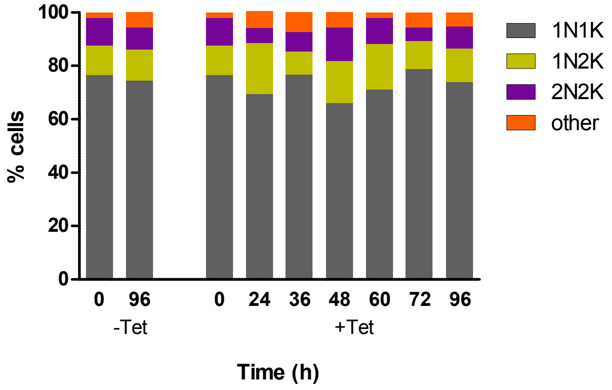

Tb927.6.2840
RIO2

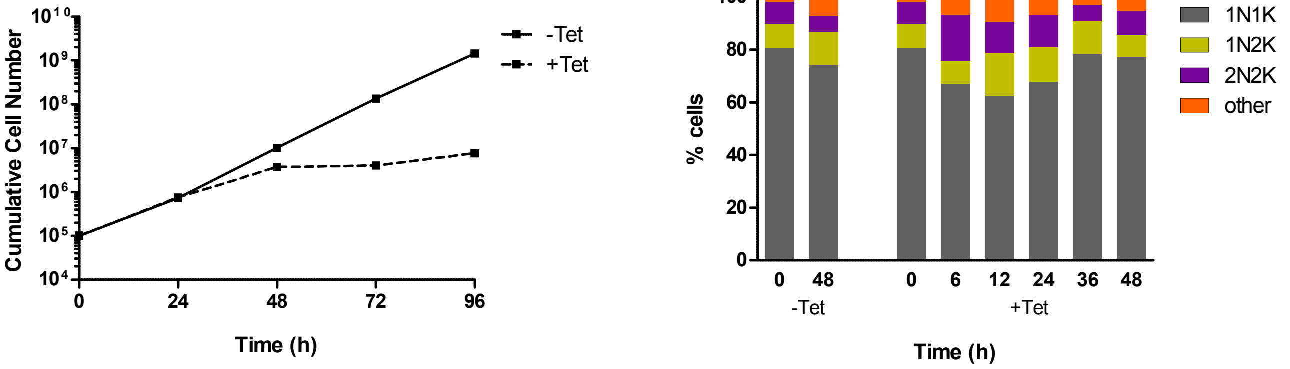

E

Tb927.10.4940
PK50

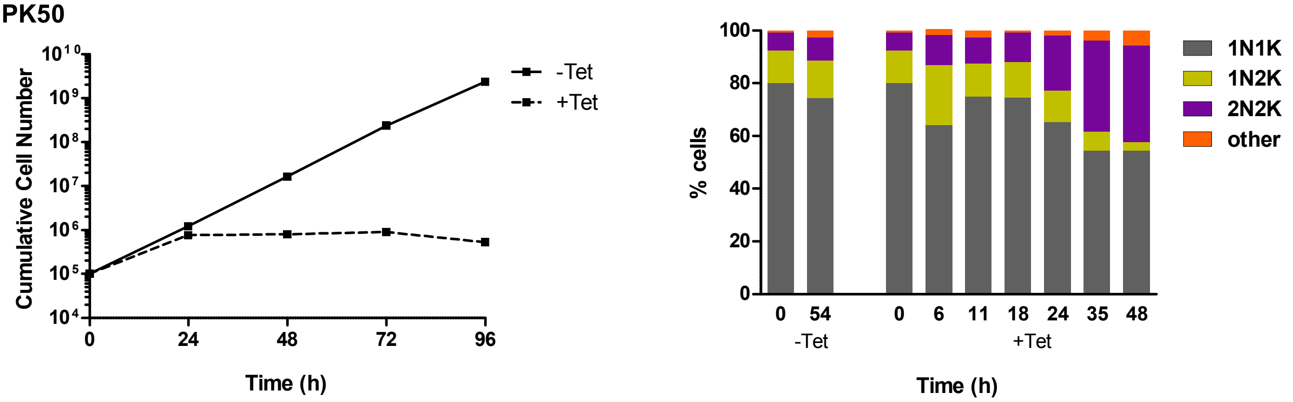

Tb927.7.6220

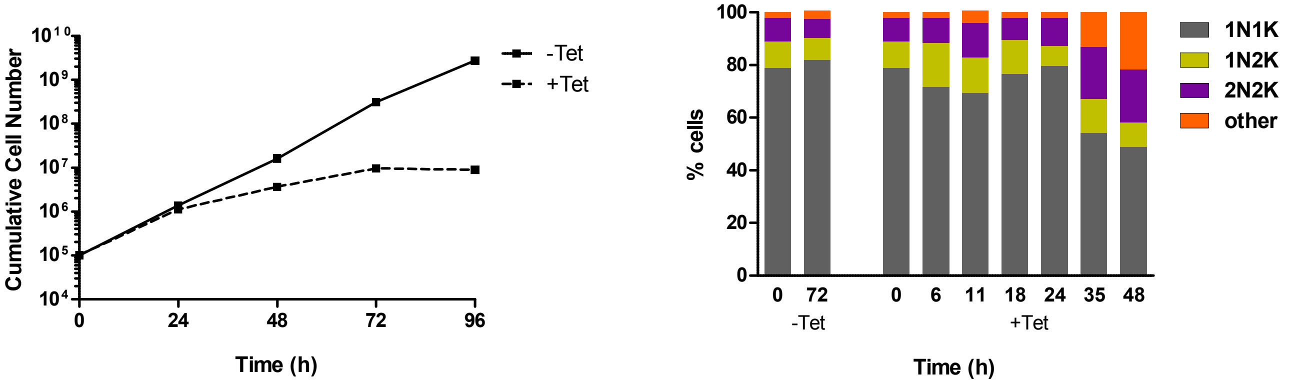

Tb927.10.4990
CRK3
Clone 1

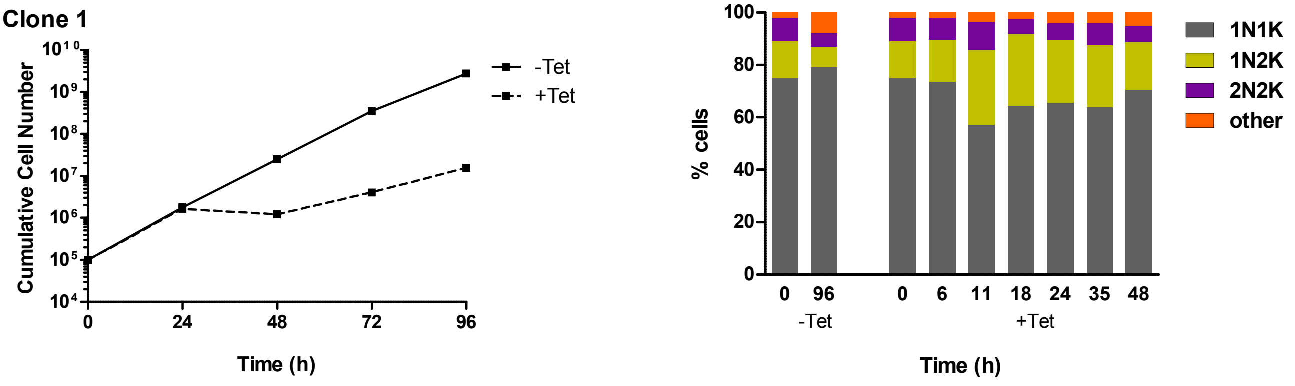

Tb927.10.4990
CRK3
Clone 2

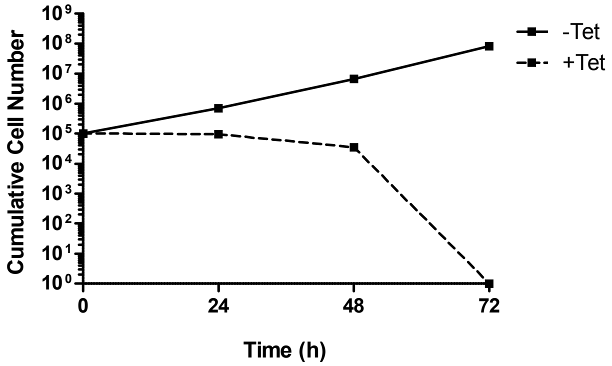

Tb927.10.13780
GSK-short

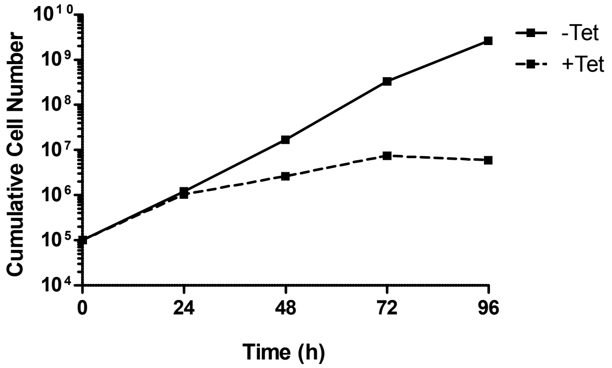

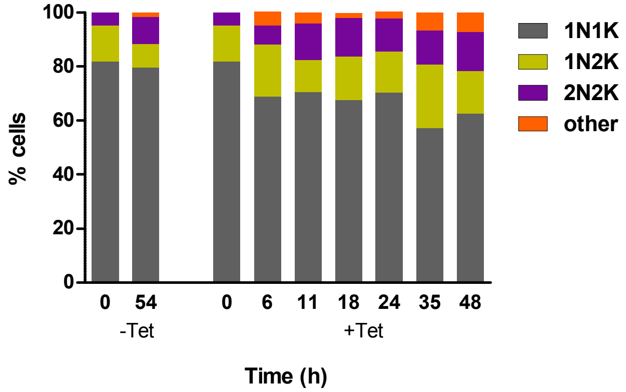

Tb927.9.14430
CK2A1

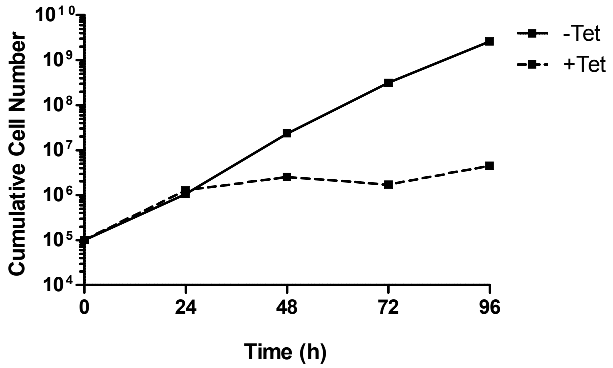

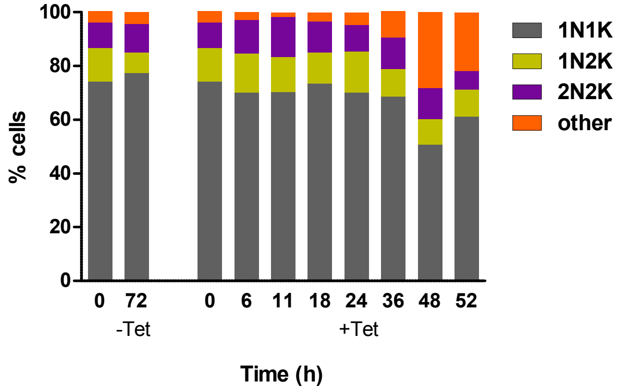

Tb927.11.4470

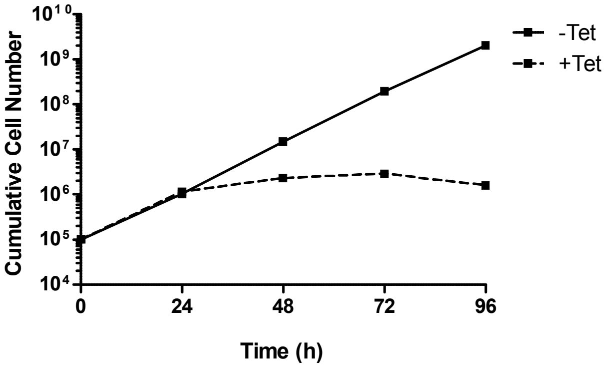

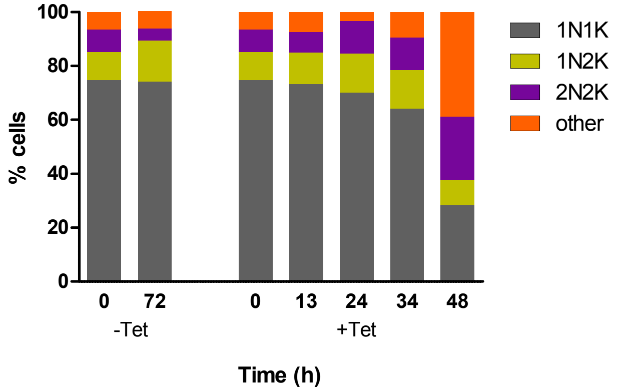

Tb927.11.14680
ATR
Clone 2

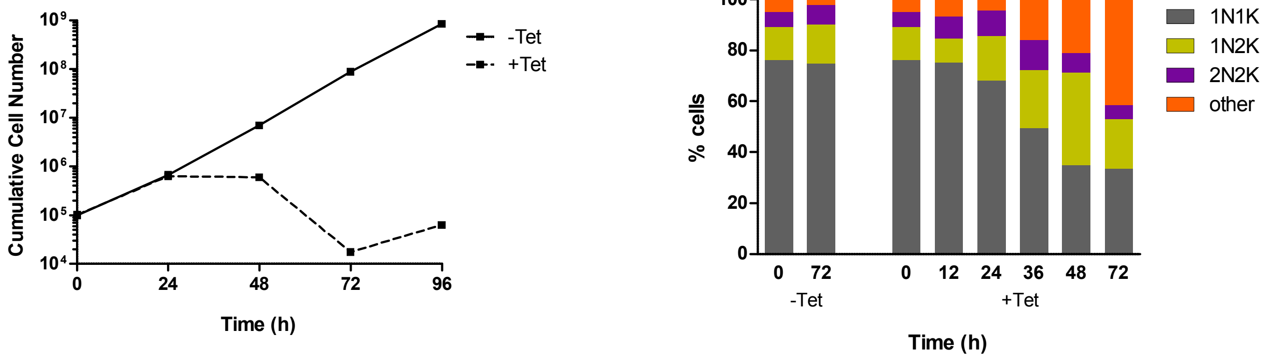

F

Tb927.11.12310
CRK12

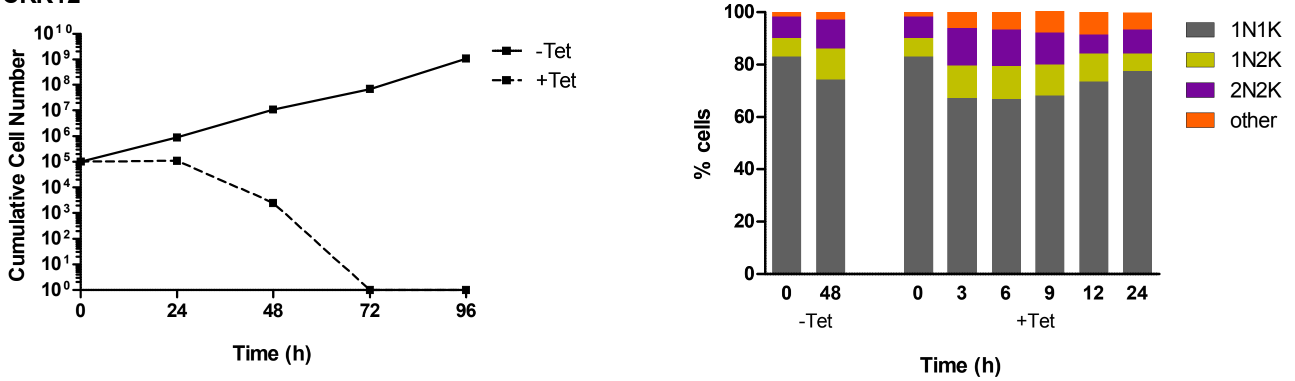

Tb927.10.2040

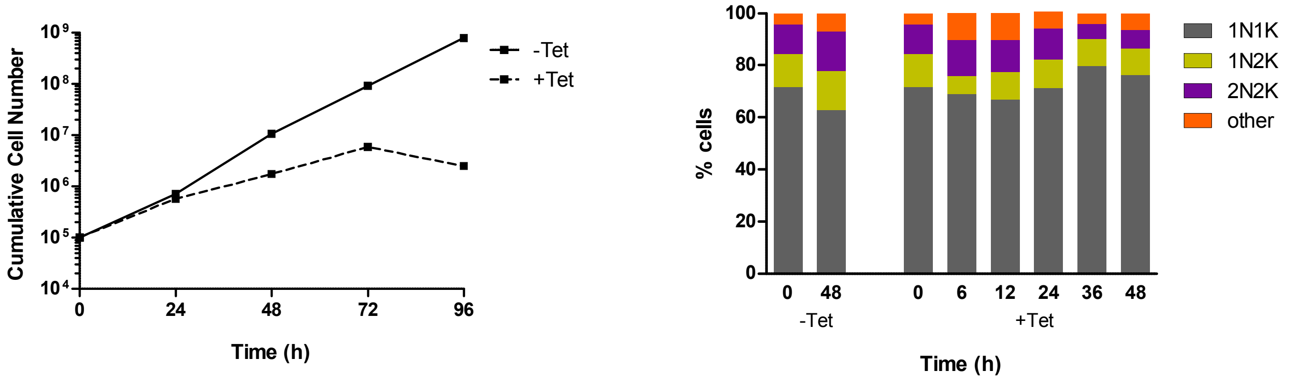

Tb927.4.420
TOR2

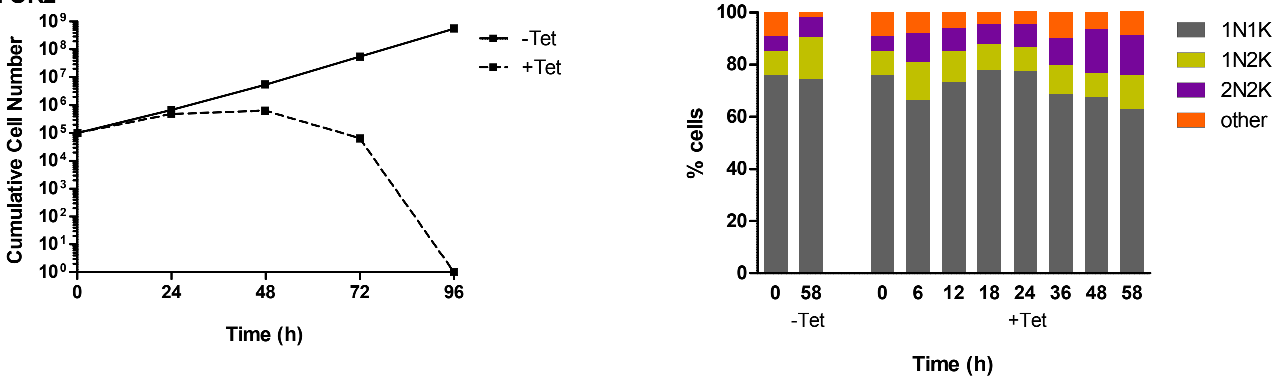

G

Tb927.3.2440

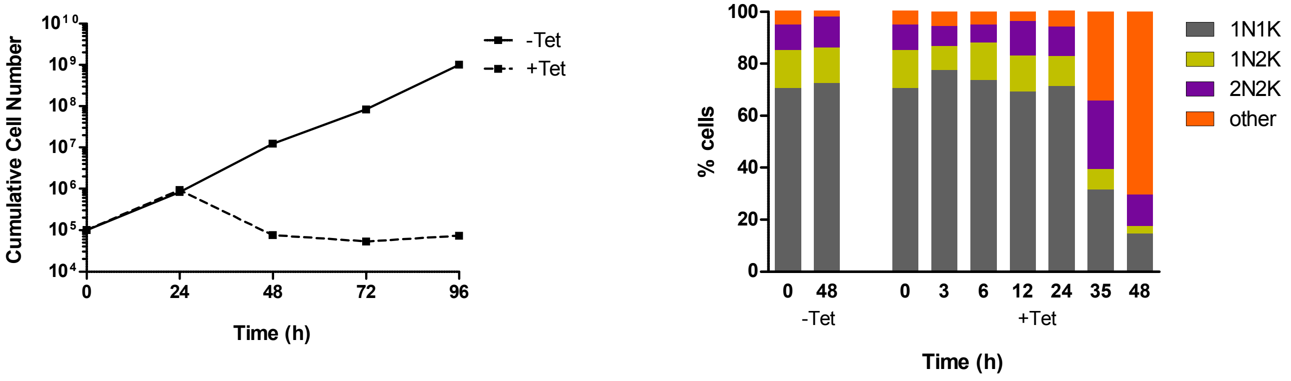

Tb927.5.790 & Tb927.5.800
CK1.1&CK1.2

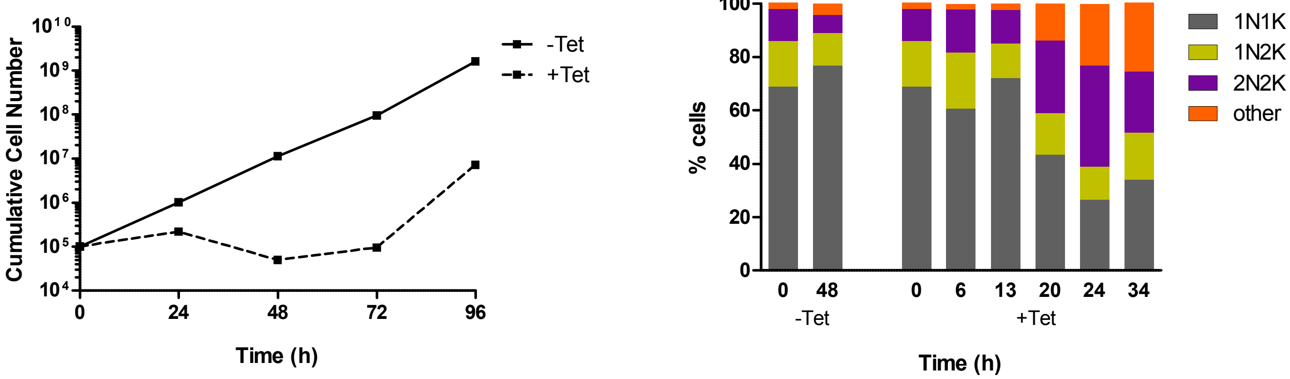

Tb927.2.4510
CRK9

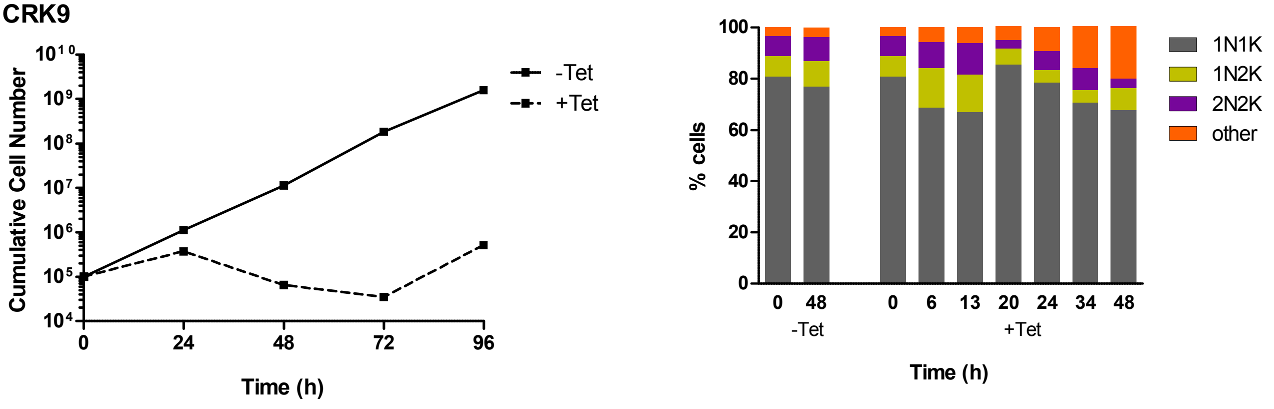

Tb927.11.12410
CLK1
Clone 2

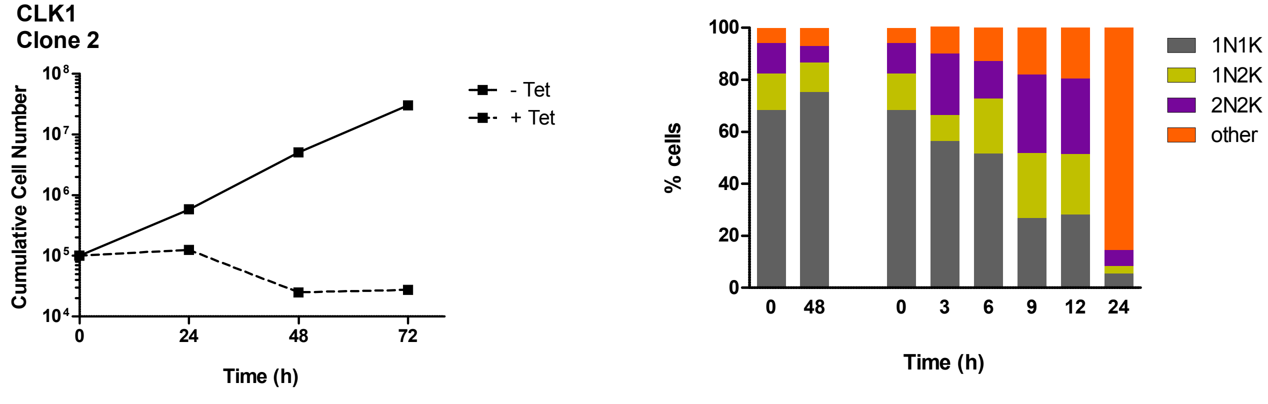

Tb927.10.5140

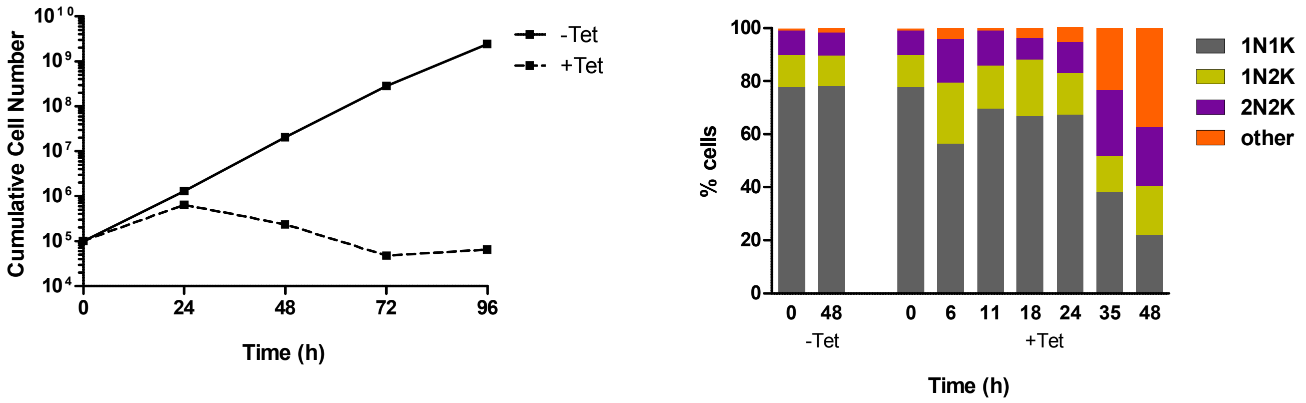

Tb927.3.690

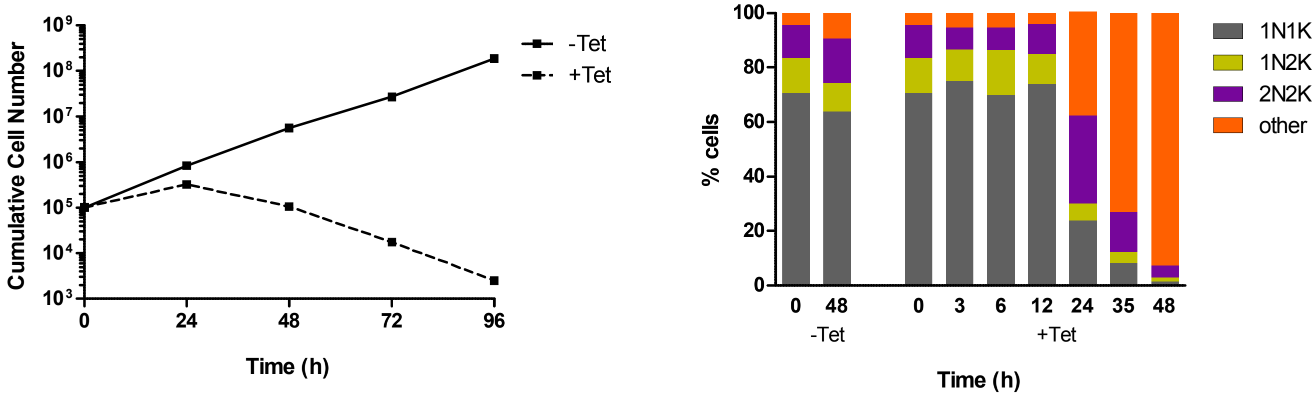

Tb927.11.8220
AUK1

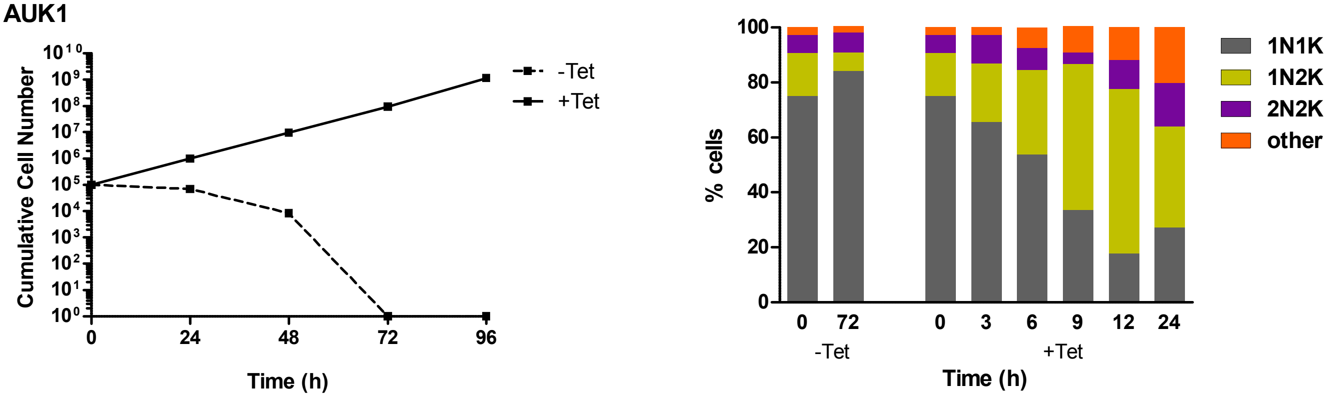

Tb927.4.5310
NEK12.2/RDK2

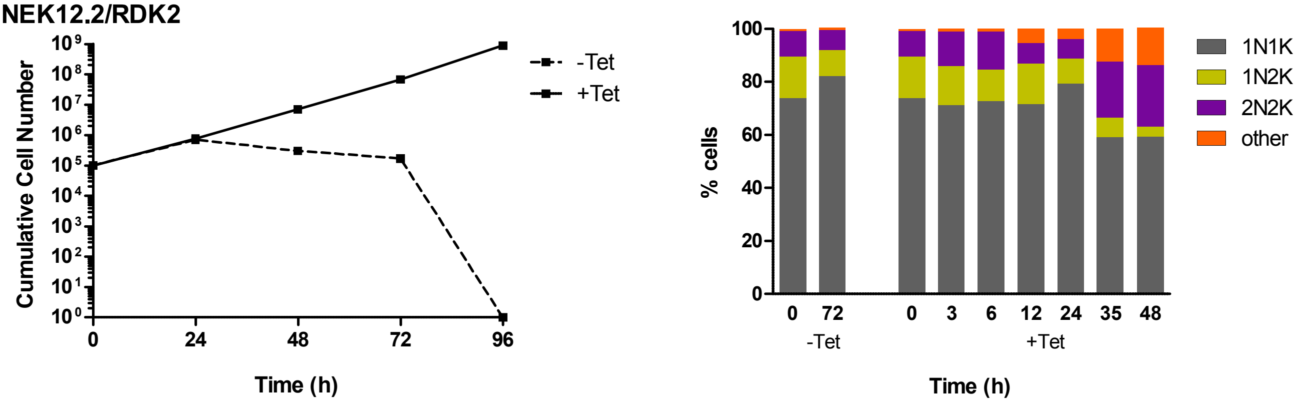

Tb927.4.5180&Tb927.8.7220
TLK1&TLK2

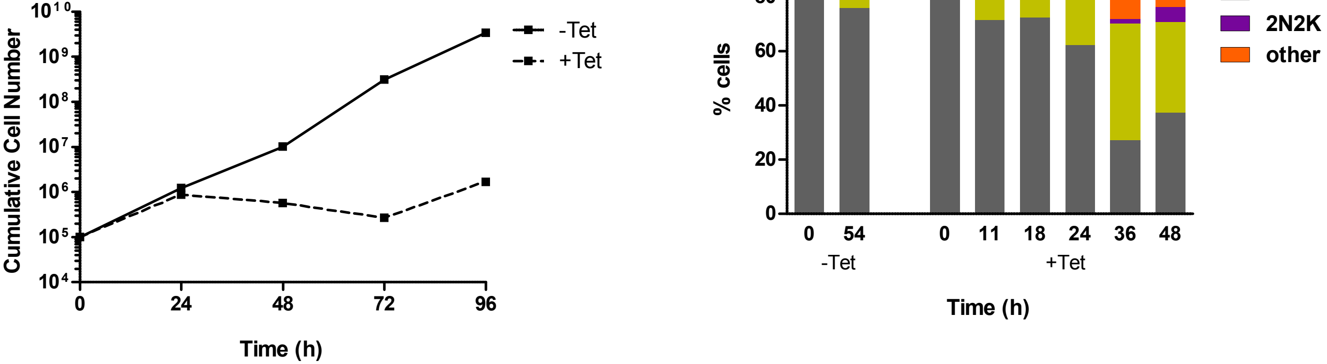

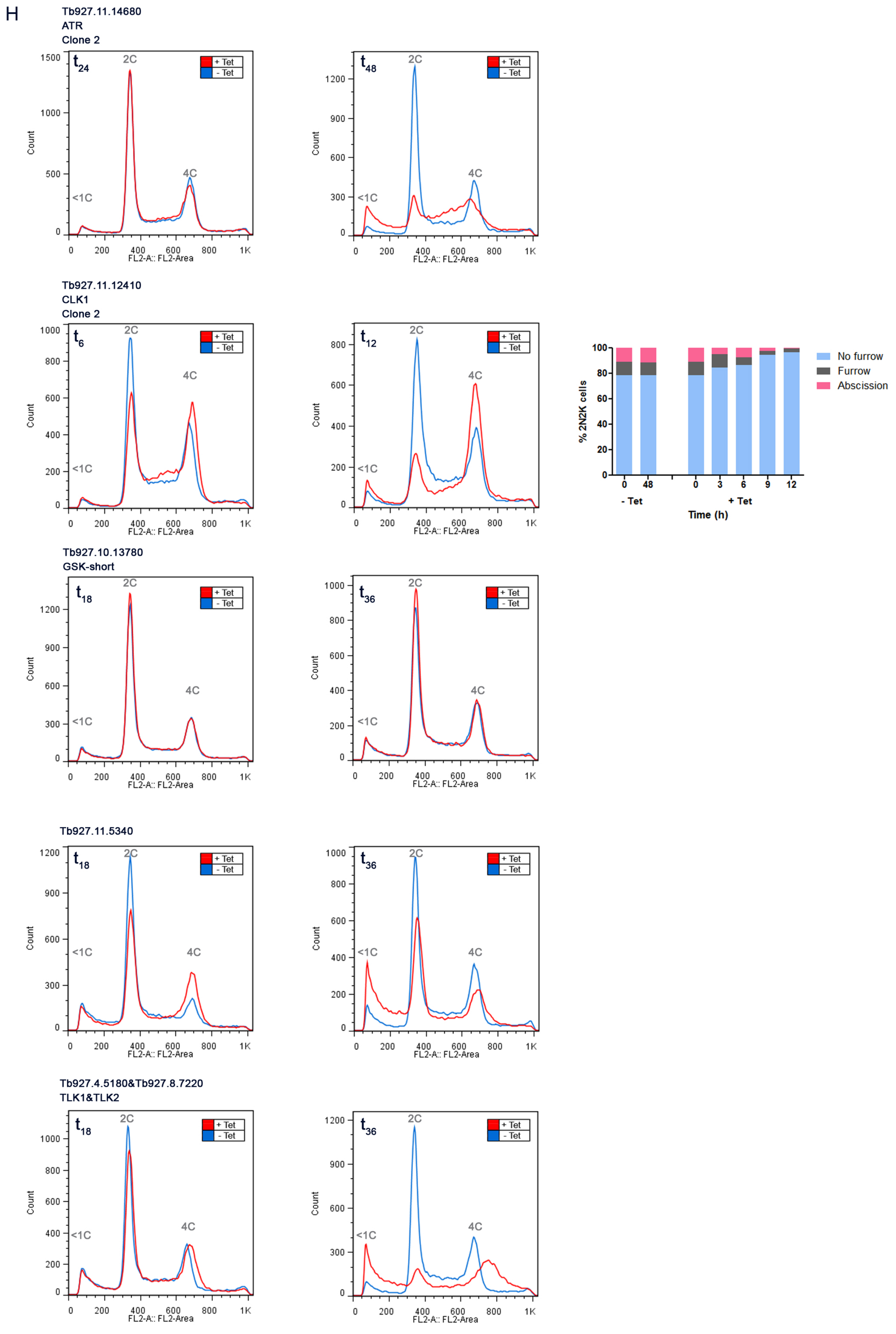

H
Tb927.11.14680
ATR
Clone 2
t24
t48
Tb927.11.12410
CLK1
Clone 2
t6
t12
Tb927.10.13780
GSK-short
t18
t36
Tb927.11.5340
t18
t36
Tb927.4.5180&Tb927.8.7220
TLK1&TLK2
t18
t36
2C
4C
<1C
+ Tet
- Tet
Count
FL2-A:: FL2-Area
% 2N2K cells
No furrow
Furrow
Abscission
- Tet
+ Tet
Time (h)
